# Commercialization of obstetric and neonatal care in the Democratic Republic of the Congo: A study of the variability in user fees in Lubumbashi, 2014

**DOI:** 10.1371/journal.pone.0205082

**Published:** 2018-10-10

**Authors:** Abel Mukengeshayi Ntambue, Françoise Kaj Malonga, Michèle Dramaix-Wilmet, Tabitha Mpoyi Ilunga, Angel Nkola Musau, Charles Matungulu Matungulu, Karen D. Cowgill, Philippe Donnen

**Affiliations:** 1 Unité d’Epidémiologie et de Santé de la mère, du nouveau-né et de l’enfant, École de Santé Publique, Université de Lubumbashi, Lubumbashi, RDC; 2 Centre de recherche en Epidémiologie, Biostatistiques et recherche clinique, École de Santé Publique Université Libre de Bruxelles, Brussels, Belgium; 3 School of Interdisciplinary Arts and Sciences, University of Washington Tacoma, and Department of Global Health, University of Washington, Seattle, Washington, United States of America; 4 Centre de Recherche en Politiques et systèmes de santé-Santé internationale, École de Santé Publique Université Libre de Bruxelles, Brussels, Belgium; Federal University of Sergipe, BRAZIL

## Abstract

**Objective:**

In the Democratic Republic of the Congo, insufficient state financing of the health system produced weak progress toward targets of Millennium Development Goals 4 and 5. In Lubumbashi, almost all women pay out-of-pocket for obstetric and neonatal care. As no standard pricing system has been implemented, there is great variation in payments related to childbirth between health facilities and even within the same facility. This work investigates the determinants of this variation.

**Methods:**

We conducted a cross-sectional study including women from admission through discharge at 92 maternity wards in Lubumbashi in March 2014. The women’s payments were collected and validated by triangulating interviews of new mothers and nurses with document review. We studied payments related to delivery from the perspective of women delivering. The total was the sum of the payments linked to seeking and accessing care and transport of the woman and companion. The determinants were assessed by multilevel regression.

**Results:**

Median payments for delivery varied by type: for an uncomplicated vaginal delivery, US$45 (range, US$17–260); for a complicated vaginal delivery US$60 (US$16–304); and for a Cesarean section, US$338 (US$163–782). Vaginal delivery was more expensive at health centers than in general referral hospitals or polyclinics. Cesarean sections done in corporate polyclinics and hospitals were more expensive than those done in the general referral hospitals. Referral of delivering women, use of more highly trained personnel, and a longer stay in the maternity unit contributed to higher expenses. A vaginal delivery in the private sector was more cost-effective than in the public sector.

**Conclusion:**

To guarantee universal coverage of high-quality care, we suggest that the government and funders in DRC support health insurance and risk pool initiatives, and introduce and institutionalize free mother and infant care.

## Introduction

The Bamako Initiative recommended direct payment for care by households as a way to fill the gap in healthcare sector budgets in several low-income countries [1–4]. However, this was not followed by measures to protect users from excessive and sometimes fixed costs of services [5]. In nearly all countries in sub-Saharan Africa, close to two thirds of the budget for maternal, newborn, and child care comes from households via direct payment made as a condition of receiving services. In these countries, more than 90% of women who have access to care must pay out of pocket [6,7].

More recently, the Abuja Accords recommended that African governments allocate at least 15% of their national budget to the healthcare sector to reduce the burden of health care expenses on households [5]. However, as this is not adhered to in most of these countries, household contributions have remained the principal source of financing of the healthcare system [6]. Given that these countries have mostly poor populations, these contributions have become a real barrier to accessing care [7–17].

Of the many studies that have investigated factors that influence household payments in this direct-pay context, few have focused on obstetric and neonatal care [14,18–34]. The majority of those were conducted from the provider perspective, and evaluated costs to health care facilities (HCF) [20–22,35]. The available evaluations from the user or society perspective are more focused on the composition of payments [14,19,24–27,31,32,34,36] than on their determinants. They have shown that, even if the relative importance of each component differs from one setting to another, and from one type of delivery to another, the components were similar: fees for drugs, the hospital stay, medical and nursing interventions, supplies, equipment, and laboratory tests. Available studies [29,30] that have looked at determinants of cost in these settings have shown that the type of delivery, distance between the user’s home and the health care facility, and the socioeconomic level of households determined the variability in delivery costs. While likely to influence the costs of care, systemic factors like the distinctive characteristics of each health zone (HZ), the type and sector of health care facilities, the type and training of the provider and the participation of the community—in terms of household adaptation to self-pay fees for care—have not yet been studied as determinants of these payments.

In the Democratic Republic of the Congo (DRC), direct payment for care by households represents 41% of health-care expenditures [37], while poverty affects more than 70% of the population [38]. The inadequacy of financing for the health-care system by the state is one of the main reasons for poor progress towards the achievement of the Millennium Development Goals (MDG), specifically MDG 4 & 5 [39]. In Lubumbashi, the second most populous city in the DRC, emergency obstetric and neonatal care (EmONC) is not subsidized; both general and maternal-infant healthcare are covered only by direct out-of-pocket payments by households at the point of care [39]. More than 90% of women attend prenatal consultations (PNC) at least once during their pregnancy and deliver in a facility [40]. While almost all of these women pay out-of-pocket, they judge the fees to be high. More than a quarter of women who delivered without attending PNC (stated that was financially unattainable for them [20].

With insufficient financing of the healthcare system by the state, the role of the private sector has increased to the point that more than 60% of HCF in Lubumbashi are private [41]. Weak regulation of the private sector in general, and of obstetric and neonatal care in particular [39]goes hand-in-hand with large variability in the charges imposed on women, and consequently in the number of women who use these services.

In this context of minimal regulation, the profit motive promoted commercialization of obstetric care characterized by competition and price wars between HCF [39], which provoked high variability in the prices of care between, and even within HCFs, for women who presented with similar conditions. This apparently arbitrary variability conveys the lack of equity that characterizes care in this setting [42–45] in which providing appropriate care to mothers and infants depends on their ability to pay.

As solidarity initiatives (mutual health, health insurance) in the area of health care increase in the country [37,46], having information about what determines payments for obstetric care and what factors explain its variability can inform measures to regulate fees for deliveries and determine the fraction of spending priorities related to childbirth [23,47,48]. Thus, this study was conducted with the objective of determining the factors explaining the variability of payments borne by households for facility-based childbirths in Lubumbashi. To do this, we ascertained the payments for facility-based childbirths, their components, and investigated the factors that influence their variability.

## Material and methods

### Research setting

The city of Lubumbashi has an area of 747 km^2^ and an estimated population, in 2014, of more than 2 million, for a density of 2,543 inhabitants per km^2^. Nearly 70% of its population lives on less than US$1 per day [38]. It is subdivided into eleven HZs, each of which has a general referral hospital (GRH), on average, 15 health centers (HC). One HZ, Kowe, does not have a GRH. In the two-level health care system in DRC, the health centers (HC) are the first level of contact with the population; the GRHs are the first level to which the HCs refer complicated cases. GRHs can in turn refer cases to the highest level, a provincial hospital like the Jason Sendwe Hospital or the University Clinics of Lubumbashi. In practice, this hierarchy is not always respected by users.

There are more than 350 HCFs in Lubumbashi (hospitals, polyclinics, and HCs), and 70% of them are urban. The private sector represents more than 60% [41]. Nearly 180 of the HCF in Lubumbashi provide maternity services [49]. In 2012, the percentage of deliveries with skilled attendance at Lubumbashi was 94%, but the rate of Cesarean sections was low, at 4.5% [40].

Until 2005, the United Nations Population Fund (UNFPA) supported the maternity ward at the general provincial referral hospital, Sendwe, by supplying drugs, supplies, and equipment for emergency obstetric and neonatal care (EmONC), though they did not manage payments to personnel. Since 2005, no other organization has stepped in to subsidize maternity care. The price of obstetric care is covered by private or parastatal companies for women whose partners work for those companies, but these women account for no more than 1% of those who deliver in Lubumbashi. Occasionally, a charitable organization or politician may pay the fees for obstetric care for insolvent women.

**Fig 1:**
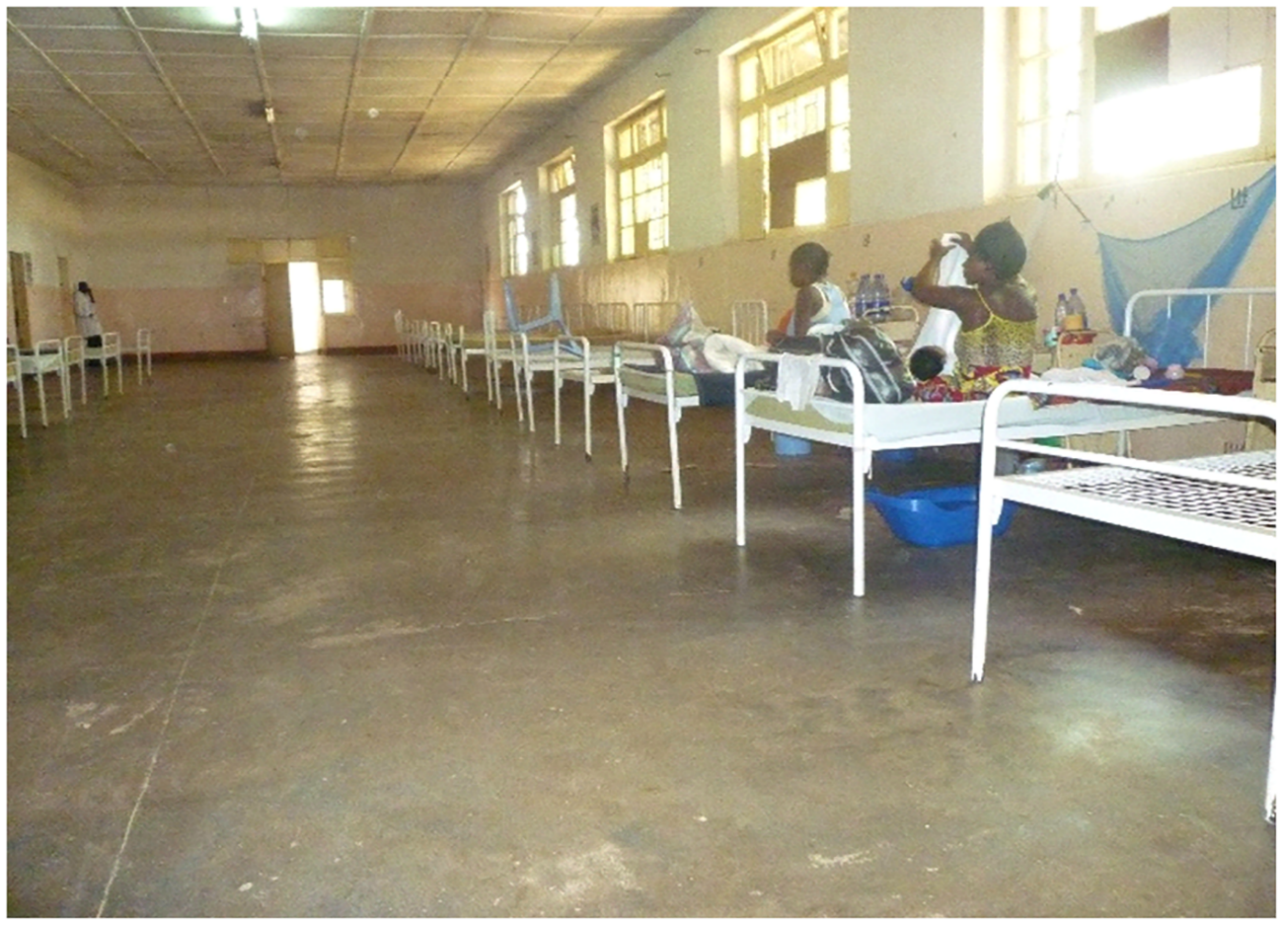
Postpartum room for VD at the Sendwe maternity ward (November 2014).

The provincial hospital, Sendwe, which formerly had close to 9,000 childbirths a year, now has only 1,600 (Fig 1; S1 Table; S1 Fig). The University Clinics of Lubumbashi (UCL) is a university hospital center for the training of doctors and nurses under the management of the University of Lubumbashi. It is a tertiary-level referral hospital. However, because of the precarity of its funding, it does not function to the full potential of its activities. Due to the lack of an appropriate public GRH in its HZ, it also serves as the GRH for the Lubumbashi HZ. Since 2010, the average annual number of deliveries there is 1200. The two parastatal hospitals, GCM-Sud and the one belonging to the Société Nationale des Chemins de Fer du Congo (SNCC), are also two large health care facilities in Lubumbashi. In addition to care provided to employees of these parastatal companies, they offer care to the general population. The number of deliveries in 2014 at these facilities was, respectively, 1800 and 900. GCM-Sud is the GRH for the Mumbunda HZ and SNCC is the GRH for the Tshamilemba HZ.

### Population

For this study, we included all the HZ, and in each of them, all HCFs that performed at least 25 deliveries in the month before the survey (March 2014). These facilities were officially recognized as providing maternal health care according to the provincial division of health’s data. Facilities not included in this study were not yet officially recognized as providing maternity care, but had occasional deliveries without having set up the appropriate services ahead of time. In each of the 92 HCF that fit the inclusion criteria, we targeted all the women admitted to the maternity ward during the study period, but only those capable of communicating with the researchers (by speaking French or at least one of the local languages: Swahili, Tshiluba, Lingala or Kikongo) and who agreed to participate in the study were retained. In all, 1404 were admitted to maternity units during this period; all were eligible for our study.

The women who read and understood French signed a written consent after having read it; for those who could not read French, the consent form was read and explained to them by the researcher in a local language, in the presence of a witness who was not a member of the study team. They then signed the consent for after confirming that they had satisfactorily understood its contents. Data were kept confidential. This study was approved by the medical ethics committee of the University of Lubumbashi (CEM-UNILU: UNILU/CEM/010/2011).

### Study

We conducted a cross-sectional study, including women admitted to the selected HCF from March 1–8, 2014. We followed the women from admission to discharge. During this period, we tracked their payments [50]. The last women followed up were discharged from the study in May of the same year. Thirty trained researchers collected data via interviews and document review.

The structured questionnaire (S1 Text) collected data on payments made and the reason for each payment, as well as sociodemographic and economic information not included in maternity records. We interviewed the head of the maternity unit to determine for each parturient which items were covered by the payments. We reviewed each woman’s medical record to collect data about her obstetric situation (reasons for admission, complications, type of delivery and care received), and reviewed invoices for fees related to delivery.

Payments made were followed daily during the whole length of the stay, by tallying receipts held by the women or their relatives, or from the maternity records when the receipt was not available [50]. Payments were validated after triangulating information i) from the parturient or family, ii) in the maternity record, and iii) by confirmation of the payment by the head of the maternity unit. For medications purchased outside of the HCF, we tallied the purchase receipts; if the receipt was missing, we took the woman’s word, provided that the medicine had been identified or that the information had been validated by the birth attendants. This was also the case for laboratory examinations or blood bought at the blood bank. If the total of the payments declared by the woman was higher than that stated by the maternity unit team and proofs of payment were not available, the difference was included in the category "other payments", which included any gratuity given to the health staff [50]. For drugs, we took into account those prescribed and purchased during the facility stay whether the woman was to continue taking them at home or not. For example, during data collection, we noted that women received prescriptions before discharge, and staff ensured that the women had obtained these drugs before discharge. We did not take into account any drugs bought outside the HCFs after the woman’s discharge.

For the women whose care was subsidized by an organization, standard pricing was used to determine the price of care and the charges covered by the payments. Payments associated with round-trip transport, when utilized, were determined based on the woman’s or a family member’s statement. If these were paid by a third party, they were estimated by taking into account the going rate for the trip. For public transportation, this total was multiplied by the number of companions accompanying the woman upon her arrival at the maternity ward. For the trip home, since we were following the women, we asked them what means of transport they would take. For those who had a personal means of transport, we estimated the price based on private taxi fare to the woman’s home. For those who took public transport, we likewise estimated the price as a function of distance. We used the same estimate whether the women planned to take a shared taxi or a minibus.

### Management and analysis of data

Data were double-entered in Excel. We studied payments for care from the perspective of women admitted to maternity units over the course of the study period. Household expenses include direct and indirect costs. In this study, direct costs comprise direct medical costs (obstetric and neonatal care: medicine, equipment, delivery, Cesarean section, episiotomy, bandages, laboratory tests, newborn care, stay, and medical record) and direct non-medical costs (transport of the woman). Indirect costs were additional payments by companions of the women for trips home to get food and clothing, and time lost by companion for supervision and stay). The total payments for delivery were the sum of payments related to: i) obstetric and neonatal treatments (direct medical costs) as well as transport of the woman (direct non-medical costs), and ii) transport of the companion (indirect costs) [50].

Time lost was estimated in terms of daily lost revenue, calculated according to the current occupation of the woman and companion as declared the day of the survey. Given the difficulty of obtaining data about household income, we utilized data from household surveys in Lubumbashi. Daily income was the quotient of the monthly income divided by 28 days. The lost income was the product of the daily income and the number of days in the maternity ward. For the women for whom housework was the only occupation, the average monthly income used to calculate the time lost was US$45 [50]. We did not include in the calculation the expenses related to the purchase of the layette (clothing for the mother and newborn). Likewise, intangible effects such as stress and emotions related to delivery were not included in the calculation of expenses. Also, given that the HCFs did not provide food for the women during their stay and the women ate their usual household food, we did not include payments for this food in our calculations. On the other hand, payments for the companion’s trip to the get the food were included. All of the expenses were expressed in US dollars (920 Congolese francs = US$1).

We used the usual descriptive statistics (percentage, mean and standard deviation, median, minimum and maximum) to describe the profile of the facilities and the women included in the study. The Mann-Whitney and Kruskal-Wallis tests were used to compare median expenses according to the characteristics of the women. The Bonferroni correction was used for pairwise comparisons of expenses by type of delivery: uncomplicated vaginal delivery (UnVD) versus complicated vaginal delivery (CVD); UnVD versus Cesarean; CVD versus Cesarean) [51]. Complicated delivery was defined according to WHO criteria as “medical problems associated with obstetric labor, such as hemorrhage (antepartum and postpartum), obstructed labor, postpartum sepsis, placenta previa, placental abruption and premature rupture of membranes, complications of abortion, severe pre-eclampsia and eclampsia, ectopic pregnancy and ruptured uterus or others. These complications can affect the well-being of the mother, the fetus, or both [52].

#### Investigation of determinants

The determinants of the variability of payments related to childbirth were investigated by forward stepwise multilevel nonlinear modeling [53] at a significance threshold of 5%. The four levels considered were: 1) the women delivering; 2) the staff managing the delivery; 3) the types of HCFs; and 4) the sector to which the HCF belonged. In this study, we considered any HCF created as a HC as a HC, regardless of the evolution of its technical platform. HCs in which, for example, cesarean sections were carried out, were included in the HC category. All sociodemographic variables, the type of delivery, the length of stay, whether a patient was referred, the person paying for care, were variables linked to the women delivering. For each type of delivery, we presented four models—empty, with the variables linked to the women delivering, with the systemic variables, and with all variables—to study the change in measures of variation of systemic variables with regard to individual variables. All analyses were performed in Stata v13.1.

#### Exploration of alternatives

After identifying the determinants of the variability in payments, we explored the most cost-effective alternative in terms of access to EmONC for vaginal deliveries [54]. We defined EmONC as “services for the treatment of direct obstetric complications that arise during pregnancy and childbirth. They include nine signal functions: i) administer parenteral antibiotics; ii) administer uterotonic drugs; iii) administer parenteral anticonvulsants for preeclampsia and eclampsia; iv) manually remove the placenta; v) remove retained products; vi) perform assisted vaginal delivery; vii) perform basic neonatal resuscitation; viii) perform surgery and ix) perform blood transfusion” [55]. In this study, we evaluated the efficacy of each option in terms of the woman’s access to signal functions i-vi of EmONC. We considered any woman who had an obstetric need and who received an appropriate intervention for that need according to the indications for each EmONC signal function as having received EmONC. In the case of VD, every woman should receive at least one EmONC signal function (injection of an uterotonic to prevent post-partum hemorrhage). We did not conduct this cost-effectiveness analysis for cesarean deliveries, since they did not respond to the same efficacy criteria as vaginal deliveries [52]. We carried out the choice of alternatives based on one factor of entry into the health care system: the ownership sector. We asked the question, for women, which of the choices, between delivering vaginally in public or parastatal HCFs (option_1) or in private or religious HCFs (option_2) was more cost-effective. In option_1, women had the choice to deliver in a public or parastatal HCF, which was a HC, GRH, Sendwe, UCL, or a parastatal facility (GCM-Sud, SNCC). In this facility, was the delivery managed by a midwife or by a physician, a generalist or a specialist, and did she receive EmONC or not? In option_2, women were exclusively in a private or religious HCF, where the options for their management were the same as those in option_1.

To answer this question, we calculated the incremental cost-effectiveness ratio (ICER) as the relationship of the difference in payments linked to vaginal birth in option_1 and option_2 to the difference in access to EmONC between the two options.

To measure the uncertainty in our cost-effectiveness model, we considered a threshold of acceptable payments—willingness to pay—of US$460 (2015 per capita gross domestic product of the DRC) [56]. We categorized the options studied according to whether they were very cost-effective (ICER < 1 x per capita GDP); cost-effective (ICER ≥ 1 x and ≤ 3 x per capita GDP); and not cost-effective (ICER > 3 x per capita GDP [57]. To test the robustness of our conclusions, we carried out a probabilistic sensitivity analysis. We created probability distributions for all the parameters of the model. For the costs, we used the payment data observed in this study. We created 100 samples using Monte Carlo simulations and calculated their expected values. We then calculated the proportion of samples that had a good cost-effectiveness for each alternative. These analyses were performed in TreeAge Pro 2018 R1.1 (Watertown, MA, USA).

## Results

Out of all the women admitted to maternity units during our study (n = 1,404), we excluded 14: 6 had left the unit within seven hours of delivery, 2 died immediately postpartum, and 6 experienced perinatal deaths (4 stillbirths and 2 neonatal deaths on the day of delivery) and refused to participate in the interview and follow-up. Only 1,390 were followed during their stay in the maternity unit.

### Recruitment sectors, health zones, and health care facilities

In order of frequency, these 1,390 women were recruited in the HZ of Lubumbashi (17.6%), Kampemba (15.7%), Katuba (12.5%) and Ruashi (12.4%). The HZ in Kenya, Mumbunda, Tshamilemba, Vangu, Kisanga and Kamalondo were, together, represented by 41.7% of the women. More than half (53.8%) were recruited at HCs, a quarter (25.1%) at GRHs, 13.3% at polyclinics, and 3.9% (54) at Sendwe and at UCL. More than half (58.9%; n = 819) were recruited from facilities in the private sector, 33.9% (471) from the public sector, and 7.2% (100) in parastatal corporate HCF (GCM-Sud and SNCC).

### Sociodemographic profile of the women

Table 1 shows that the mean age of the women was 26 (standard deviation 6 years; minimum = 14; maximum = 46); 11.9% were less than 20 years old, while 10.3% were over 35 years. The majority were married (95.2%). For the most part (70.1%), they had attended secondary school, but 18.7% had only primary education, and 0.2% had not attended school. The principal occupation of these women was homemaking (53.2%), but 25.9% were also vendors, and 20.9% had other occupations such as civil service. The median number of deliveries was 3 (min = 1, max = 12); 22.9% were delivering for the first time, while 23.4% were at their fifth or higher delivery. The majority (92.5%; n = 1,286) had made at least one PNC visit during their pregnancy. The median frequency of prenatal visits per woman was 3 (minimum = 1; maximum = 7), but only 45.7% (588) completed the number of recommended visits (4 visits).

**Table 1:** Sociodemographic characteristics of women delivering at health care facilities in Lubumbashi, DRC, 2014.

	Number (1,390)	Percentage	Mean ± Standard Deviation
Age (years)			26 ± 6
< 20	165	11.9	
20–34	1,082	77.8	
≥ 35	143	10.3	
Marital status			
Married	1,323	95.2	
Single	67	4.8	
Education			
Primary	260	18.7	
Secondary	974	70.1	
Post-secondary and University	156	11.2	
Occupation			
Homemaker	739	53.2	
Vendor	360	25.9	
Other occupations	291	20.9	
Parity (number)			
1	319	22.9	
2–4	746	53.7	
≥ 5	325	23.4	
Attendance at prenatal care	1,286	92.5	
*Frequency*			
*1*	*79*	*6*.*2*	
*2–3*	*619*	*48*.*1*	
*≥ 4*	*588*	*45*.*7*	

Table 2 shows that 65 women (4.7%) were referred to GRH or to Sendwe by other HCF. Among the 1,390 women included in the study, 1,270 (91.4%) delivered vaginally, while 120 (8.6%) had Cesarean sections. During childbirth, 381 women (27.4%) had complications. In order of frequency, obstructed delivery (8.9%), placenta previa, placental abruption or premature rupture of membranes (7.6%), ante- and postpartum hemorrhage (4.4%), eclampsia (3.2%), and uterine rupture (2.5%), were the principal maternal complications observed. In total, 261 women (68.5%) of the 381 who had complications had a VD, while 31.5% underwent a Cesarean. The majority of VDs (92.0%) were led by a nurse or midwife; only 6.1% (77) were by a general practitioner and 1.9% (24) by a specialist. For the Cesarean (120), 70.0% (84) were performed by a general practitioner and 30.0% (36) by a specialist.

**Table 2:** Admission characteristics of women delivering at health care facilities in Lubumbashi, DRC, 2014.

	Number (n = 1,390)	Percentage
Referral	65	4.7
Type of delivery		
UnVD	1,009	72.6
CVD	261	18.8
Cesarean section	120	8.6
Complications		
*Obstructed delivery*	*124*	*8*.*9*
*Other complications*‡	*105*	*7*.*6*
*Ante- and postpartum hemorrhage*	*61*	*4*.*4*
*Uterine rupture*	*34*	*2*.*5*
*Eclampsia*	*44*	*3*.*2*
Service provider for VD		
Nurse or midwife	1,169	92.0
General practitioner	77	6.1
Specialist	24	1,9
Service provider for Cesarean-section		
General practitioner	84	70.0
Specialist	36	30.0
Accompanied to the maternity ward	403	29.0
Relationship of companion		
*Mother of the woman*	*154*	*38*.*2*
*Sister-in-law*	*83*	*20*.*6*
*Sister*	*63*	*15*.*6*
*Mother-in-law*	*53*	*13*.*2*
*Friend*	*37*	*9*.*2*
*Spouse*	*13*	*3*.*2*
Principal occupation of the companion		
*Homemaker*	*247*	*61*.*3*
*Vendors or civil service agents*	*156*	*38*.*7*
Person who paid		
Couple	1,210	87.1
Insurance subscriber	111	8.0
Relative	69	5.0

^‡^: placenta previa, placental abruption and premature rupture of membranes

In total, 403 women (29.0%) were accompanied to the maternity ward by someone close to them. This person was either her mother (38.2%), sister-in-law (20.6%), sister (15.6%), mother-in-law (13.2%), friend (9.2%) or spouse (3.2%). The principal occupation of these companions was homemaking (61.3%), but 38.7% (156) were vendors or civil service agents. Most women (87.1%) paid for their care themselves (with their partners); 8% of women were subsidized by their husband’s employer, while the fees of 5% were paid by family members.

### User fees for deliveries in Lubumbashi

Table 3 shows a median payment of US$45 (minimum = 17, maximum = 260) for an UnVD; US$60 (minimum = 16, maximum = 304) for a CVD; and US$338 (minimum = 163, maximum = 782) for a Cesarean. The components of this payment were similar between the different types of deliveries. For each, obstetric and neonatal treatments represented nearly 90% of the total of expenses paid by households. The median payment for transport was higher for a Cesarean section (US$7) than either a complicated (US$4) or UnVD (US$4). Still, transportation expenses were a larger share of the total expenses for uncomplicated (8.2%) and complicated (7.4%) VD than for Cesareans (3.2%; p < 0.001). Likewise, the proportion of payments for the purchase of supplies and medicine, the hospital stay, other fees and transport was significantly higher in the case of a Cesarean than in the case of other types of deliveries (p < 0.001).

**Table 3:** User fees in US$ of deliveries and their components in Lubumbashi, DRC, 2014.

Expenses	Expenses in US$ for VD						Expenses in US$ for Cesarean-section (n = 120)			p (Bonferroni)		
	Uncomplicated (n = 1,009)			Complicated (n = 261)								
	*Ave*.*±SD*	*%*	*Med* *(Min-Max)*	*Ave*.*±SD*	*%*	*Med* *(Min-Max)*	*Ave*.*±SD*	*%*	*Med* *(Min-Max)*	*UVD/CVD*	*UVD/C*	*CVD/C*
**Total per household**^**a**^	**53.6 ± 29.0**		**45 (17–260)**	**69.9 ± 44.5**		**60 (16–304)**	**353.6 ± 94.9**		**338 (163–782)**			
*Recourse and access to care*^*b*^	*51*.*8 ± 28*.*3*	*96*.*6*	*44 (17–260)*	*65*.*8 ± 42*.*3*	*94*.*3*	*53 (16–304)*	*326*.*4 ± 87*.*3*	*92*.*3*	*308 (144–690)*	*< 0*.*001*	*< 0*.*001*	*< 0*.*001*
**Obstetric and neonatal care**^**c**^	**47.4 ± 27.1**	**88.4**	**39 (16–254)**	**60.7 ± 41.2**	**87.0**	**49 (16–300)**	**315.1 ± 86.5**	**89.1**	**300 (135–686)**	**< 0.001**	**< 0.001**	**< 0.001**
Medical Record	2.6 ± 1.2	4.9	2 (1–8)	2.2 ± 1.3	3.6	2 (1–8)	3.0 ± 1.5	0.9	3 (1.1–11)			
Delivery and care of newborn	33.4 ± 21.4	62.4	28 (4–177)	42.1 ± 33.2	61.4	33 (8–252)	206.3 ± 76.2	58.3	190 (82–450)			
Equipment & medicine	3.6 ± 2.9	6.7	2 (2–57)	5.9 ± 4.4	7.2	3 (2–28)	6.0 ± 3.4	1.7	56 (6–158)			
Hospital stay	6.2 ± 6.4	11.5	4 (2–57)	7.4 ± 6.7	10.5	5 (2–57)	40.4 ± 19.7	11.4	36 (16–190)			
Other fees	1.5 ± 3.2	2.8	1.2 (1–42)	2.2 ± 5.4	4.0	1.6 (0–33)	58.6 ± 13.6	16.6	28 (0–100)			
**Transport of parturient**^**d**^	**4.4 ± 5.2**	**8.2**	**4** (2–68)	**5.2 ± 6.1**	**7.4**	**4** (2–59)	**11.3 ± 13.9**	**3.2**	**7 (2–70)**	**0.13**	**< 0.001**	**< 0.001**
*Transport of companion*^*e*^	*1*.*8 ± 4*.*5*	*3*.*3*	*1*.*2 (0–37)*	*4*.*0 ± 1*.*3*	*5*.*7*	*2*.*0 (0–138)*	*27*.*3 ± 22*.*3*	*7*.*7*	*19 (1–92)*	*0*.*001*	*< 0*.*001*	*< 0*.*001*

Ave: average; a = b+e; b = c+d; UnVD/CVD: UnVD in relation to CVD; UnVD/C: UnVD in relation to Cesarean; CVD/C: CVD to Cesarean; SD: standard deviation

Table 4 shows that payments for delivery varied according to the ownership sector of the HCF (p < 0.001) and, in each sector, between types of HCF by level of the health care system (p < 0.001). We noted that women who had delivered in public HCs paid less than those who delivered in HCs in other sectors. On the contrary, relative to HCs of other sectors, it was in the public HCs that payments for cesarean sections were higher. Women who delivered in public GRHs paid less than those who delivered in parastatal hospitals. Payments linked to VD were lower for women who delivered in religious polyclinics compared to those who delivered in private polyclinics. There was no statistically significant different in payments for cesareans between women who delivered in public GRHs and those who delivered in parastatal or religious hospitals compared to those who delivered in private polyclinics.

**Table 4:** User fees in US$ of deliveries according to the profile of sector and health facilities, Lubumbashi, DRC, 2014.

Sector	All	Health centers^1^		GRH		Sendwe		CUL		Polyclinics		*p*^†^^#^
	N	N	Med (US$) (Min-Max)	N	Med (Min-Max)	N	Med (US$) (Min-Max)	N	Med (US$) (Min-Max)	N	Med (US$) (Min-Max)	
Public	471											
UnVD		84	39 (24–109)	173	44 (17–116)	32	53 (38–75)	31	102 (95–186)			*<0*.*001*
CVD		22	45 (16–142)	45	60 (25–161)	12	59 (49–98)	14	112 (30–161)			*<0*.*001*
C-Section		8	351 (317–492)	31	287 (168–474)	10	322 (275–442)	9	334 (245–380)			*0*.*13*
Parastatal	100											
UnVD				63	70 (41–126)							
CVD				22	72 (60–219)							
C-Section				12	359 (267–567)							
Private	685											
UnVD		422	42 (20–115)							89	56 (24–207)	*<0*.*001*
CVD		95	48 (26–124)							33	69 (29–304)	*<0*.*001*
C-Section		21	331 (278–424)							25	422 (199–782)	*<0*.*001*
Denominational	134											
UnVD		79	47 (20–127)							25	33 (28–53)	*<0*.*001*
CVD		14	57 (30–108)							10	37 (29–121)	*0*.*33*
C-Section		3	318 (287–323)							3	632 (381–642)	*0*.*04*
*p(UnVD)*^‡^^#^		*<0*.*001*		*<0*.*001*				*<0*.*001*		*<0*.*001*		
*p(CVD)*^‡^^#^		*<0*.*001*		*<0*.*001*				‡		*0*.*06*		
*p(C-Section)*^‡^^#^		*0*.*012*		*0*.*12*				‡		*0*.*11*		

Med: median;

^‡^: comparison of median expenditures for each type of delivery between different sectors;

^†^: comparison of median expenditures for each type of delivery between the different health facilities;

^#^: Kruskal-Wallis;

^1^: Certain HC performed Cesareans without being authorized to do so.

Within sectors, we noted that in the public sector, payments were also variable between types of HCFs. For vaginal deliveries, they were lower in HCs and GRHs compared to Sendwe and UCL. Although the difference in payment for cesarean was not statistically significant, payments tied to this type of delivery were lower in the GRHs compared to Sendwe, UCL, and the HCs. In the private sector, whatever the mode of delivery, payments were higher in polyclinics than in the HCs. In the religious sector, the variability in payments was not statistically significantly different between HCF for CVD. However, they were for uncomplicated vaginal birth and cesarean section. Payments for VD were higher in HCs compared to polyclinics, and those for cesarean were higher in polyclinics compared to HCs.

As far as the HZs (Table 5), there was similarly variability between payments for deliveries between HCs in different zones (p < 0.001) and, within each, between the HCFs (p < 0.001). For vaginal deliveries, the HCs in Vangu and Katuba had lower payments compared to those of other HZs. Although the payments for cesareans were also variable among HCs, this difference was not statistically significant. In the GRHs, Kampemba, Mumbunda, Ruashi, and Kamalondo had the highest payments for VDs among the HZs. In the polyclinics, only payments for VD differed among HZs; they were higher when women had delivered in polyclinics in the Lubumbashi HZ than when they delivered in other HZs. Although the difference in payments for cesarean among HZs was not statistically significant, it was likewise in the polyclinics of the Lubumbashi HZ that they were higher.

**Table 5:** User payments in US$ of deliveries according to of Health zone profile and type of health care facility, Lubumbashi, DRC, 2014.

Health Zones	All	Health centers^1^		GRH		Sendwe		CUL		Polyclinics		*p*^†^^#^
	*N*	*N*	*Med* (US$) *Min-Max*	*N*	*Med* (US$) *Min-Max*	*N*	*Med* (US$) *Min-Max*	*N*	*Med* (US$) *Min-Max*	*N*	*Med* (US$) *Min-Max*	
Kamalondo	67											
UnVD		24	52 (30–84)	22	51 (41–83)							*0*.*72*
CVD		12	65 (50–122)	5	52 (45–52)							*0*.*04*
C-Section		2	354 (347–361)	2	276 (265–286)							*0*.*02*
Kampemba	218											
UnVD		88	42 (27–75)	49	64 (35–79)					20	53 (33–117)	*<0*.*001*
CVD		24	45 (34–71)	19	67 (51–161)					5	49 (42–54)	*<0*.*001*
C-Section		5	331 (290–347)	8	458 (419–567)							*<0*.*001*
Katuba	174											
UnVD		95	37 (23–71)	34	45 (32–116)					16	43 (36–89)	*<0*.*001*
CVD		18	42 (26–63)	4	66 (46–154)					2	54 (43–65)	*0*.*02*
C-Section		2	329 (308–350)	3	274 (163–324)							*0*.*31*
Kenya	128											
UnVD		77	40 (24–78)	24	42 (37–51)							*0*.*19*
CVD		10	62 (42–112)	4	62 (47–63)							*0*.*69*
C-Section		5	355 (317–388)	8	264 (237–323)							*0*.*001*
Kisanga	76											
UnVD		39	36 (26–68)	29	39 (37–44)							*0*.*18*
CVD		7	43 (34–48)	1	60							
Lubumbashi	245											
UnVD		43	62 (25–127)			32	53 (38–75)	31	102 (95–186)	37	148 (46–260)	*<0*.*001*
CVD		17	65 (27–124)			12	59 (49–98)	14	112 (30–161)	22	167 (66–304)	*<0*.*001*
C-Section		4	335 (318–356)			10	322 (275–442)	9	334 (245–380)	14	457 (199–782)	*<0*.*001*
Mumbunda	128											
UnVD		50	40 (27–78)	36	81 (66–126)							*<0*.*001*
CVD		17	44 (29–142)	10	83 (66–219)							*<0*.*001*
C-Section		7	338 (320–492)	8	356 (331–458)							*0*.*96*
Ruashi	173											
UnVD		100	41 (20–76)	8	53 (35–64)					25	33 (28–55)	*<0*.*001*
CVD		12	53 (34–70)	3	64 (59–75)					8	36 (29–70)	*0*.*03*
C-Section		4	309 (278–397)	10	297 (270–366)					3	379 (376–381)	*0*.*13*
Tshamilemba	91											
UnVD		31	61 (20–115)	27	59 (39–72)							*0*.*55*
CVD		11	86 (30–137)	12	68 (48–81)							*0*.*34*
C-Section		3	297 (283–298)	7	390 (318–483)							*0*.*03*
Vangu^¥^	90											
UnVD		30	19 (26–32)	6	33 (17–111)					24	40 (24–70)	*0*.*20*
CVD		11	24 (16–31)	6	39 (25–79)					7	43 (29–60)	*0*.*01*
C-Section				4	231 (168–247)					2	242 (158–266)	*0*.*32*
*p(UnVD)*^‡^^#^		<0.001	<*0*.*001*			<*0*.*001*						
*p(CVD)*^‡^^#^		<*0*.*001*	<*0*.*001*			<*0*.*001*						
*p(C-Section)*^‡^^#^		*0*.*29*	<*0*.*001*			*0*.*12*						

Med: median;

^‡^: comparison of the median expenditures for each type of delivery between the different HZ;

^†^: comparison of median expenditures for each type of delivery between the different health facilities;

^#^: Kruskal-Wallis;

^1^: Certain HC completed Cesareans without being authorized to do so;

^¥^: in ZS Ruashi, Polyclinic represents Ruashi Military Hospital

In Kamalondo, there was no difference in payments for VD between the HCs and the GRHs. Conversely, CVD and cesareans were more expensive in the HCs than in the GRHs. In Kampemba and Katuba, whatever the mode of delivery, women paid more delivering in the GRHs than in the HCs and the polyclinics. In the Kenya HZ, cesarean section at the HC entailed more payments than at the GRH. In Kisanga, there was no difference in payments for delivery between the HCs and the GRHs. This HZ did not record any cesareans during the study period. In the Lubumbashi HZ, regardless of the mode of delivery, payments were different among HCFs. Women who delivered at Sendwe paid less than those who delivered in other HCFs, including GRHs. For VD, delivering at UCL and in the polyclinics was more expensive for women, while for cesarean section, polyclinics were more expensive than other HCFs. In both Mumbunda and Ruashi, VD were more expensive in the GRHs than in the HCs. Delivering vaginally cost less for women in the polyclinics in Ruashi. In Tshamilemba, only cesarean was expensive if performed in polyclinics rather than HCs. There was no statistically significant difference in the variability of payments for VD between HCs, GRHs, and polyclinics in this HZ. In Vangu, only CVDs cost more in polyclinics compared to the GRH and HC.

Table 6 shows that, regardless of the type of delivery, referral increased the median payments for delivery (p < 0.001). Complications during the course of a VD also influenced the variability of the payments. Apart from the treatment of postpartum infections, management of eclampsia, obstructed labor and post-partum hemorrhage led to an increase in payments (p < 0.001). For Cesareans, it was placenta previa, placental abruption, and uterine rupture, as well as eclampsia, which led to higher payments (p < 0.001).

**Table 6:** User fees in US$ of deliveries according to the demographic and obstetric profile of the expecting women, Lubumbashi, DRC, 2014.

Variables	VD (n = 1,270)			Cesarean section (n = 120)		
	*N*	*Median in* US$ *(min-max)*	*p*#	*N*	*Median in* US$ *(min-max)*	*p*#
Origin of the woman			< 0.001			< 0.001
Initial consultation	1237	46 (17–304)		88	324 (163–782)	
Referral	33	61 (16–219)		32	379 (242–632)	
Type of delivery			< 0.001			
UnVD	1009	45 (17–260)				
CVD	261	60 (16–304)				
EmONC for			< 0.001			0.04
None	1022	45 (17–260)				
Eclampsia	21	66 (31–304)		23	347 (208–782)	
Obstructed labor	58	64 (26–237)		66	335 (168–567)	
Postpartum Hemorrhage	45	62 (16–199)		16	313 (249–419)	
Other complicationsʰ	90	52 (18–271)		15	422 (163–642)	
Postpartum infections	34	48 (18–104)		ɸ		

^#^: Mann-Whitney or Kruskal-Wallis;

^ʰ^: placenta Previa, placental abruption, and uterine rupture;

^ɸ^: managed as sequela, so no additional fees

In Table 7, payments for delivery increased with increasing specialization of the service provider though this was not statistically significant, given the small number of deliveries attended by physicians (p = 0.2). Cesareans performed by specialists incurred statistically significantly higher payments than those performed by a generalist (p < 0.001). This table also shows that payments were higher when they were covered by a third party rather than paid directly out of pocket at the point of care (p < 0.001).

**Table 7:** User fees in US$ of deliveries according to the profile of the service provider, the duration of the stay and the source of financing for care, Lubumbashi, DRC, 2014.

Variables	VD (n = 1,270)			Cesarean-section (n = 120)		
	*N*	*Median in* US$ *(min-max)*	*p*#	*N*	*Median in* US$ *(min-max)*	*p*#
Service provider			0.2			< 0.001
Nurse/Midwife	1169	46 (16–304)				
General practitioner	77	55 (23–172)		84	323 (163–632)	
Specialist	24	60 (38–105)		36	362 (245–782)	
Duration of stay (days)			< 0.001			
1–3	990	46 (17–304)				
4–7	255	49 (16–260)				
≥8	25	66 (29–219)		120	338 (163–782)	
Person who paid			< 0.001			0.07
Couple	1107	46 (16–304)		103	338 (163–782)	
Relative	60	47 (19–219)		9	320 (168–376)	
Subscriber	103	68 (21–260)		8	513 (267–642)	

^#^: Kruskal Wallis

### Determinants of the variability of payments related to deliveries in Lubumbashi

The results of the multilevel nonlinear model (Table 8) showed that among factors identified by the univariable analysis, only the type of HCF in which the women delivered, referral of the parturient, and the source of the financing for care were the common determinants of the variability of the expenses of both vaginal and cesarean deliveries. In addition to these factors, the type of delivery and length of stay were particular determinants of this variability for VD (Table 8). Health sector did not affect payments for either vaginal or cesarean deliveries. The credentials of the service provider affected expenses for cesarean section.

**Table 8:** Coefficients of the variables that determine payments for deliveries in US $, Lubumbashi, DRC, 2014: Multilevel nonlinear model.

Factors	Empty Model		Individual Model		Contextual Model		All factors Model	
	Coef.	95%IC	Coef.	95%IC	Coef.	95%IC	Coef.	95%IC
**Vaginal delivery**								
PNC (Yes/No)			7.8	2.6 to 13.1**			7.8	2.5 to 13.1**
CVD vs UnVD			9.2	5.6 to 12.7**			9.0	5.5 to 12.6**
Referral vs non referral			13.1	4.0 to 22.3**			12.8	3.6 to 21.9
Duration of stay(days)				**				**
4–7 vs ≤ 3			1.7	-1.9 to 5.3			1.6	-2.0 to 5.2
≥ 8 vs ≤ 3			16.8	8.1 to 25.5			16.8	8.1 to 25.5**
Sources of financing				**				**
Couple vs Subscriber			2.1	-4.6 to 8.9			2.0	-4.7 to 8.8
Relatives vs Subscriber			14.2	8,5 to 20.0			14.2	8.5 to 20.0
Health Sector						$		$
Corporate vs public					27.8	-12.8 to 68.3	21.2	-17.1 to 59.5
Private vs public					-28.4	-63.1 to 6.2	-25.2	-58.0 to 7.6
Denominational vs public					-42.3	-89.0 to 4.4	-41.1	-85.2 to 3.1
Type of HCF						*		*
GRH vs HC					-12.7	-51.6 to 26.2	-14.6	-51.4 to 22.2
Sendwe vs HC					-10.2	-57.8 to 37.4	-9.6	-54.6 to 35.3
UCL vs HC					28.4	-15.8 to 72.7	26.4	-15.4 to 68.2
Polyclinic vs HC					56.4	19.9 to 92.8	52.3	17.8 to 86.7
Service provider						$		$
General practitioner vs N-M					13.0	0.7 to 25.4	9.9	-2.6 to 22.3
Specialist vs N-M					10.8	-8.8 to 30.5	8.4	-11.3 to 28.1
**Random-effects Parameters**	Esti		Est		Esti		Esti	
*Health Sector*: *Identity [var(_cons)]*	*0*.*0*	*0*.*0*			*0*.*0*		*0*.*0*	*0*.*0 to 146*
*HCF*: *Identity [var(_cons)]*	*1416*	*591 to 3394*	*1197*	*499 to 2873*	*474*	*163 to 1376*	*412*	*134 to 1268*
*Provider*: *Identity [var(_cons)]*	*141*	*38 to 525*	*117*	*29 to 471*	*64*	*7 to 576*	*70*	*8 to 582*
*var(Residual)*	*687*	*636 to 744*	*632*	*584 to 684*	*688*	*636 to 745*	*632*	*585 to 684*
**Cesarean-Section**								
Sources of financing				**				**
Couple vs Subscriber			-21.5	-72.6 to 29.5			-16.4	-64.5 to 31.6
Relatives vs Subscriber			100.4	40.5 to 160.2			83.2	26.5 to 140.0
Referral vs non referral			44.4	14.3 to 74.5			49.4	20.8 to 78.1**
Sector						$		$
Corporate vs public					53.4	-6.3 to 112.5	2.9	-55.8 to 61.7
Private vs public					-39.9	-91.9 to 12.2	-41.5	-91.2 to 8.2
Religious vs public					16.5	-60.9 to 93.8	0.1	-73.4 to 73.6
Type of HCF						*		**
GRH vs HC					-51.3	-102.9 to 0.2	-45.7	-94.1 to 2.7
Sendwe vs HC					-26.5	-87.9 to 34.9	-26.7	-84.5 to 31.0
CUL vs HC					-91.7	-167.5 to -15.9	-106.5	-176.4 to -36.6
Polyclinic vs HC					105.4	62.8 to 147.9	94.1	54.2 to 134.1
Service provider (Specialist vs General practitioner)					57.4	18.3 to 96.4**	62.4	26.3 to 98.5**
**Random-effects Parameters**	Esti		Est		Esti		Esti	
*Health Sector*: *Identity [var(_cons)]*	*0*.*0*	*0*.*0 to 2071*			*0*.*0*		*0*.*0*	
*HCF*: *Identity [var(_cons)]*	*4107*	*1015 to 16616*	*2265*	*381 to 13461*	*0*.*0*	*0*.*0 to 0*.*02*	*0*.*0*	
*Provider*: *Identity [var(_cons)]*	*958*	*88 to 10479*	*1329*	*193 to 9167*	*0*.*0*	*0*.*0*	*0*.*0*	*0*.*0*
*var(Residual)*	*5786*	*4394 to 7621*	*46167*	*3496 to 6098*	*5384*	*41067 to 7058*	*4254*	*3303 to 5479*

Coef. = coefficients; Gp = General practitioner; N-M = Nurse-Midwife; $ = non-significant;

* = p < 0.05;

**: p < 0.001; Esti: estimations

In Lubumbashi, a VD in the polyclinics cost more for women. In giving birth in these HCFs, they paid an additional US$52.8 (95%CI: 17.8 to 86.7) compared to those who delivered in the HC. Cesareans performed in the UCL were less expensive than those performed in the HC (-US$106.5; 95%CI: -176.4 to -3.6). When performed in the polyclinics, they were more expensive than in the HC (+US$94.0; 95% CI: 54.2 to 134.1). Women who were referred paid more than those who were not (UnVD: +US$12.8; 95% CI: 3.6 to 21.9; Cesarean section: +US$49.4; 95% CI: 20.8 to 78.1). VD complicated by eclampsia or obstructed labor increased payments compared to uncomplicated deliveries (+US$9.0; 95%CI: 5.5 to 12.6). For VDs, a stay in the maternity ward greater than one week was accompanied by an increase in payments compared to a stay of three days at most (+US$16.8; 95% CI: 8.1 to 25.5). We noticed, however, that the payments for care were higher when they were paid directly at the point of care by relatives than when they were subsidized by a third party (VD: +US$14.2; 95% CI: 8.5 to 20.0; Cesarean: +US$83.2; 95% CI: 26.5 to -140.0). When the woman was operated on by a specialist, she paid US$62.4 (95% CI: 26.3 to 98.5) more than when she was operated on by a general practitioner.

### Care-seeking trajectory

**Fig 2:**
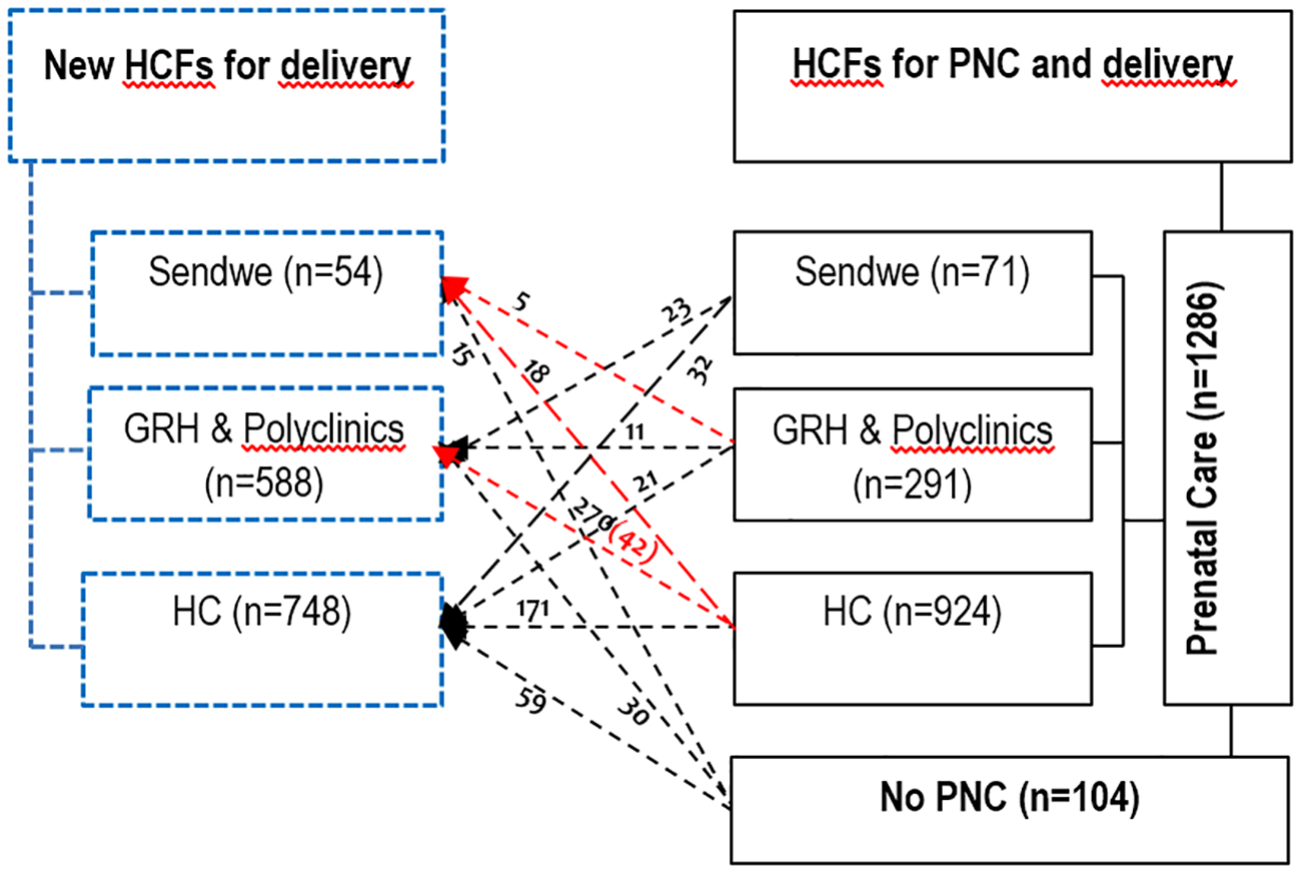
Care-seeking trajectory. The arrows and red texts represent the women who were referred to the higher level (n = 65).

Some women went to a different HCF at the time of delivery than the one where they received PNC, either because of quality of care or price (Fig 2). Among the women who went to PNC in the HC (924), 270 gave birth in the GRH and 18 at Sendwe; 171 women changed HC (gave birth in a HC other than the one attended for PNC). At the same time, 112 other women delivered at these HC: 59 of these had not attended PNC, 32 attended PNC at Sendwe, and 21 at the GRH. Among the 291 women initially attending PNC at the GRH, 5 were referred to Sendwe, and 21 gave birth at a HC; 11 women changed from one GRH to another at the time of delivery. At delivery, 270 women coming from HC, 23 coming from Sendwe, and 30 who had not attended PNC gave birth at a GRH. Among 71 women initially attending PNC at Sendwe, 23 gave birth in GRH and 32 in a HC. In total, 15 women who had not attended PNC, 5 who attended PNC at a GRH, and 18 at a HC gave birth at Sendwe. More than half of the women (56.7%) who had not attended PNC gave birth at a HC, 28.8% at a GRH, and 14.4% at Sendwe. Eleven women who attended PNC at a polyclinic gave birth in a GRH. The number of women who gave birth in the GRH was double the number of the women who attended PNC at those same HCF.

### Alternative health care system

**Fig 3:**
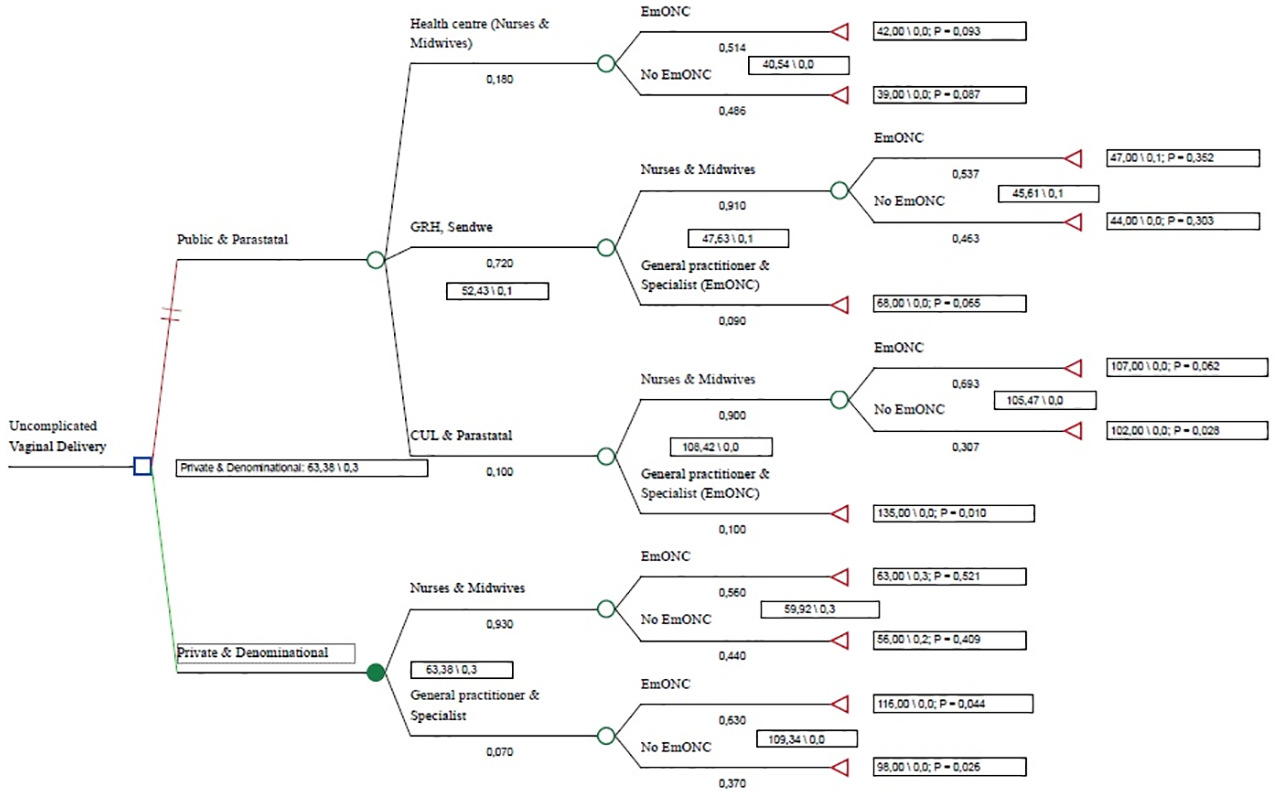
Decision tree for choice of health care sector for VD in Lubumbashi, DRC.

In terms of the relationship between providing at least one EmONC signal function and fees for care, we noted that private HCFs had higher fees for care (US$63) than public HCFs (US$52), but that three times more women had received at least one EmONC signal function at private as opposed to public or parastatal HCFs (30% vs. 10%) (Fig 3).

In terms of cost-effectiveness (Table 9), we observed that delivering vaginally in the private and religious sectors was more cost-effective for women than in the doing so in the public sector. Also, for either option (1 or 2), we observed that being attended by a nurse or midwife for a VD was more cost-effective for women than being attended by a doctor. Using a measure of extra women managed as a measure of efficacy, we found the cost of choosing to deliver in a private HCF of US$56 per direct maternal complication avoided. Using a threshold of US$460 per need covered, we observed that in all the options, the fees for VD were lower compared to the willingness to pay. But option_2 was more cost-effective. After a sensitivity analysis, this option retaine a good cost-effectiveness ratio in 81% of samples.

**Table 9:** Cost-effectiveness of vaginal deliveries: Private versus public & parastatal.

Strategy†	Cost (US$)	Incremental Cost (US$)	Effet	Incremental Effet	ICER
Public & Parastatal	52		0,065		
Private & Denominational	63	11	0,261	0,196	56

^†^: all approaches are undominated

## Discussion

### Obstetric care is expensive in Lubumbashi

In this study, we observed that in Lubumbashi, the cost of a VD is US$45 and of a Cesarean, US$338. This cost comprised payments for medical procedures, medications, transport, and other payments. Compared to the payments in other African countries reported by Perkins et al [58], those in Lubumbashi were higher. According to these authors [58], the average payments for VD in 2006 was US$4.5 in Tanzania, US$6.6 in Burkina Faso, and US$14.2 in Kenya. For delivery by Cesarean, it was US$7.7, US$13.2, and US$30.4, respectively. The payments observed in Lubumbashi seem to approach those reported by Borghi et al [50] for Benin (US$49.2) and Ghana (US$42.1), for VDs, but remain exceptionally high for Cesareans. However, if the payments for care were higher in Lubumbashi, the components were fairly similar to those in Benin and Ghana [50]. However, in Tanzania and Burkina Faso [58], transport of the parturient represented more than half of the expenses of the delivery, as compared to only close to 7% of expenses in Lubumbashi. The overall urban character of Lubumbashi, with strong accessibility to transportation, explains this difference [41], as well as the higher price paid for care.

### Why is obstetric care so expensive in Lubumbashi?

The fact that care was not subsidized explains the high price of deliveries in Lubumbashi [59]. In fact, the recorded payments in the countries mentioned previously are similar to those observed at Sendwe before 2000 (US$2–3), when 75% of the hospital’s US$6 million budget was covered by the parastatal corporation Gécamines. Due to this price, Sendwe had close to half (9,500) of the deliveries of Lubumbashi, while the other half were spread among the other parastatal HCFs whose budgets were also covered (S1–S3 Figs). This coverage of care also had a regulatory role regarding the private sector [48,60–62], in the sense that the prices of care at Sendwe and similar HCF were a benchmark for the price range in the private sector. The withdrawal of Gécamines from the financing of Sendwe (creating a budget deficit of US$4.5 million) brought about not only a drop in the annual number of deliveries in this HCF of close to 80% (S3 Fig), but also removed the regulatory role of this facility on the supply and price range of care in the private sector [62]. In this context, the necessity of recovering funds for operational expenses and staff salaries led to anarchy in the organization and provision of general and obstetric care [63].

At the organization level, we note the difficulties of structuring care packages in compliance with the primary healthcare strategy, which stipulates that for optimal functioning VDs be completed at the HCs, while the package of complementary activities be offered in the GRHs and polyclinics [39]. For example, while managing VDs at the higher-level GRHs and polyclinics increased access to facility birth for the surrounding population [46], these deliveries were managed by highly credentialed staff, whose fees were higher than those of the staff of the HC (Table 5). Thus, the price of care was higher. Likewise, an uncontrolled and unregulated medicalization of the HCs led to their providing components of care normally reserved for higher-level facilities [39,63]. This is the case with Cesareans, which were performed clandestinely in certain private and public medicalized HCs, and for which women paid more than those performed in GRHs (Table 4).

In terms of supply, the operational level of the healthcare system is characterized by competition and commercialization (commodification) of obstetric and neonatal care—particularly in the private sector—in an environment where the state has progressively lost its dominance [39]. Additionally, for a package of similar care, women spent distinctly different amounts depending on whether they gave birth in a HC, GRH, polyclinic, at UCL or at Sendwe, and whether the service provider was a midwife, a general practitioner, or a specialist, whether the treatments were subsidized or not, and whether the woman lived in the center or on the outskirts of the city.

In this study, we observed in the univariable analysis that the sector to which a HCF belonged influenced the variability in payments for care, but in the multivariable analysis, we did not note this influence. This loss of significance is due to the fact that the public sector is not only the end point of many obstetric procedures for which management began in the private sector, but also for those in which women self-referred from the private sector, hoping to find less expensive care in public HCFs. With this continuity of payments between the private and public sectors and, in looking at payments for care from the perspective of the user, these payments are often overestimated for the public sector and underestimated in the private sector [60].

Therefore, if we consider only the sector to which HCFs belong, there are multiple mechanisms at the root of the variability in payments for care. For example, in the private sector, HCFs function completely on the basis of fees for care paid by users [39]. The costs of HCFs—operational costs, personnel payments, rent, maintenance, fees dues to the HZ as well as various taxes owed to the state—are borne by users. Thus, the prices of care are fixed taking into account all these costs [39]. On the other hand, in the public sector, certain costs, notably the salary of staff and the rent are not borne by users. But just as in the private HCFs, the staff there receives commissions based on the fees paid by users, as well as hazard pay, which varies widely among categories of staff. Their essentially public ends impose a limit on the fixing of prices for certain acts, even if this limit is not always respected. In the parastatal sector, the GCM-Sud and SNCC hospitals, which used to operate from the budget of the companies to which they belong, no longer do so exclusively. Managed from here forward as units of production of services by these companies, the search for profits has likewise influenced the increase in the price of care for the population that seeks care there [64, 65]. This explains why, with the UCL, they are, of all the GHRs, the ones with the highest payments for delivery. As for the UCL, they function according to a mixed model, between that of the private, parastatal, and public HCFs, as described above. In the denominational sector, even if the search for profits exists as well, the public good leads to moderation in billing for care in this sector [39,66].

Payments for the same mode of delivery also varied among HZs and within HZs, between HCFs of different levels of the health care system. Globally, this variability is tied to the preponderance of the various sectors within each HZ [60]. For example, in the Lubumbashi HZ, where this variability in payments between HCFs was large, this is explained by the high concentration of private HCFs, particularly polyclinics where women pay more for delivery.

In most HZs where payments for VD were higher in the HCs than in the GRHs, apart from the GRH, these HCs were private. On the other hand, in those where payments were higher in the GRH than the HC, the GRH was either parastatal (Tshamilemba and GCM-Sud) or affiliated with the university (UCL). In Kisanga, where the highest-functioning HCs (registering the most monthly deliveries) are religious, payments for VD were not different between the GRH and the HCs. Sendwe Hospital is, of all the HCFs, the one in which payments for delivery were the least elevated compared to other HCFs. These low payments are explained by its current status as a public HCF, after the transfer of its management from the University of Lubumbashi to the Ministry of Public Health, and also by the fact that on that occasion, the price of delivery there had been dictated in 2012 by the governor of the province at US$20 for VD and US$150 for cesarean section. Although additional fees have been added by staff, and because of fees for transport, this amount has varied widely, but remains lower than payments observed in other HCFs.

The fact that payments for cesareans for women admitted to HCs are higher than for those at GRHs can be explained by the fact that this procedure was not always performed directly in these HCs. Instead of being referred, women admitted to these HCs were often operated on in a polyclinic that has an operating room, and then returned to the HCs for post-operative management. In this case, the fees paid by the women would have included the rental of the operating room. In the private sector, given that the limits between the activities of “medical centers” and polyclinics are poorly determined; these centers use this practice to perform cesareans. Often, they take this name to avoid the fees imposed by the state on polyclinics [39].

These higher expenses for care are, in general, a survival strategy for service providers, and, to a certain extent, for the healthcare system—all HCF allocate 5–10% of their revenue to the Central Bureau of the HZ, which in turn allocates the same percentage to the higher level [39]. Increasing the price of care is also used by the staff of public HCF as an approach to reduce work overload. At Sendwe, between 2009–2010, the price of VD was specifically increased from US$20 to 40 to reduce the daily number of deliveries, which left more free time for the staff to work in the private sector to increase their income.

In this study, we also remarked that the type of HCF in which the women delivered, referral of the parturient, and the source of the financing for care were the common determinants of the variability of the expenses of both vaginal and cesarean deliveries. The type of delivery (complicated versus uncomplicated) and length of stay were particular determinants of this variability for VD (Table 8). Occupation of the woman affected the price of VD, while the credentials of the service provider affected only the price of a cesarean.

In fact, referral increases the expenses of care on the one hand due to fees borne by delivering women for transportation between the HCF where they initially presented and the one to which they were referred [50], and, on the other hand, to persistence on the part of those in the HCs or polyclinics and then the repetition, in the referral facilities, of tests and examinations already completed at the facility that referred the patient. These data support our previous results [40]: in 2012, we observed that in Lubumbashi, additional payments related to transportation and to repetition of procedures in the referral facilities dissuaded certain women from accepting the referral.

In the case of CVD or Cesarean sections, in addition to buying medication and supplies—which are not always available in the HCFs—tips for staff or payments for monitoring of the parturient and her baby contribute to excessive payments for care [50]. Thus, the more serious the prognosis of the delivery, the more families had to pay. In this way, the financial capacity of each woman determines her management. In the private facilities, staff prefers to watch and wait instead of intervening when it comes to certain women likely to be insolvent, or they prefer surgical interventions—at times, very expensive—when a woman appears more solvent. Meanwhile, in the public facilities, women are detained at the end of their stay if they are insolvent[67].

From these observations, it is clear overall those payments for obstetric care depend very little on choices made by women themselves apart from the choice of which facility to give birth (Fig 2). In the majority of cases, they are subject to the effects of a system which is imposed on them and over which they have no control. The healthcare system meant to protect them from excessive payments for care[44] is itself in part responsible for these arbitrary abuses, because of its weak performance: poor financing, unsustainability of certain HCFs, poorly regulated or unregulated care, unregulated human resources, HCF that are insufficiently humane, but also because it survives only thanks to direct payments for care by households[39].

### Expensive, but does this care have a good cost-effectiveness ratio?

Finally, the results of this study showed that the HCFs where women paid a lot for VD–private HCFs—were also those in which they had received at least one EmONC signal function. In fact, despite the proliferation of billable procedures, and the persistence in care for cases that are complicated and should be referred, attitudes for which they are often reproached, the availability of drugs and supplies and the fact the providers are often more available there makes these HCFs attractive for the majority of women, because they complement the public sector’s gaps. Despite not having established a relationship between provision and the cost of care in the private sector, Mackintosh [60] made similar observations about excessive procedures. In demonstrating that the private sector where women paid dearly had also provided EmONC functions to more women than the public sector, our study highlights the fact that quality has a price and one cannot hope to reduce maternal and neonatal mortality without taking it on. This information shows on the one hand that, in dedicating itself to the public good, without substantial financing, the public sector sacrifices procedures essential for the survival of women and infants [63]; on the other hand, in looking solely for profit, without regulatory measures, the private sector provides some of these procedures, but in such a way as to cause catastrophic expenditures for the majority of households [9]. Also, the fact that the context of care provision in the private sector is very little regulated and controlled, in terms of who does what, where, and how and at what price, is troubling, and leads one to question its efficacy. The private sector is often the source of obstetric complications evacuated to the public sector [39].

Our analysis does not recommend that from here on, all women should deliver vaginally in the private sector, but that they should be exempted from paying for obstetric care, which would permit controlling prices in all sectors and would also even out the quality. It also underlines the fact that, in the context of protecting women from excessive expenses of care, programs to reduce the expenses of obstetric care based solely on coverage of medications and supplies without taking into account health care providers, are liable at best to have only a small impact, and at worst, to be harmful[50]. A small impact because medications represent a small percentage with respect to expenses as a whole (Table 3), and harmful because in Lubumbashi in particular, as in DRC in general, experiments with covering the expenses of medication showed that care tended to be over-billed by staff to make up for the loss of fees that they would have obtained by selling medications and supplies to parturients. The inadequately paid staff resort to this type of extortion to supplement the meager salary they receive[68]. Thus, to be more effective, any programs would have to take into account the medical and nursing workforce, medications and supplies, and transport for complicated deliveries[23].

Since this study was completed in a setting where proof of payment is not always demanded by clients, it is possible that there may be bias in the data gathered based on statements of women or service providers. The woman might have forgotten a fee paid for by a family member, while the staff might have, voluntarily, declined to mention informal charges such as gratuities[50]. Such a bias might have resulted in an underestimation of the payments made by the women. However, as mentioned in the methods section, we triangulated data between multiple sources in an effort to reduce bias. Even if such a bias existed, it would only increase the totals reported by the women. Further, having represented the distance between the HCF and the woman’s residence by transport fees is also a limitation of this study. Even if health care coverage in Lubumbashi is high, women may have chosen to deliver in a HCF near to or far from their home. This choice may have influenced the means of transport used, and consequently the fees paid.

## Conclusion

In Lubumbashi, the variation in payments for obstetric and neonatal care is associated with their commercial nature, related to the dysfunction of the health care system, which is the weak regulation of the organization and provision of care. These factors, which led to the commercialization of care and competition of health care packages among health facilities, explain the arbitrary expenses of care borne by households. Although the private-sector HCFs are cost-effective in the provision of EmONC, the price of care given in this sector might be unaffordable for households. To guarantee universal coverage of high-quality health care, we suggest to the government and funders in DRC that they support initiatives to put in place health risk pools and health insurance, which are still rare in the DRC, and that they introduce and institutionalize free maternal and infant care.

## Supporting information

S1 Table Number of health facilities with health services of the mother, the newborn and the child in Lubumbashi. (DOCX)

S1 Fig Cumulative number of new facilities offering the essential and emergency obstetric and neonatal care/treatments in Lubumbashi according to their year of creation. (TIF)

S2 Fig Number of women to the essential and emergency obstetric and neonatal care/treatments in/at Sendwe versus in other health facilities in Lubumbashi. (TIF)

S3 Fig Variation of the number of deliveries at the maternity ward Sendwe as a function of/according to the price. Coefficients of regression: -0.805; 95%IC: -0.521 to -1.00 P < 0,001.
(TIF)

S1 Text Expenses for obstetric and neonatal care in Lubumbashi: Tool for data collection. (DOC)
