# Supplementary material for: Commercialization of obstetric and neonatal care in the Democratic Republic of the Congo: A study of the variability in user fees in Lubumbashi, 2014
Source: PLoS One. 2018 Oct 10;13(10):e0205082. doi: 10.1371/journal.pone.0205082 (PMC6179261; doi:10.1371/journal.pone.0205082)
Supplement: S1 Table — (DOCX) [file pone.0205082.s001.docx]

**Table 1. Number of health facilities with health services of the mother, the newborn and the child**

| **Health Zone** | **Number of facilities** | **Percentage** |
| --- | --- | --- |
| Lubumbashi | 60 | 17.5 |
| Mumbunda | 42 | 12.2 |
| Kampemba | 22 | 6.4 |
| Kenya | 29 | 8.5 |
| Ruashi | 42 | 12.2 |
| Vangu | 2 | 0.6 |
| Kowe | 1 | 0.3 |
| Kamalondo | 4 | 1.2 |
| Kisanga | 70 | 20.4 |
| Katuba | 37 | 10.8 |
| Tshiamilemba | 34 | 9.9 |
| **Total** | **343** | **100.0** |
