# Supplementary figures and images for: Commercialization of obstetric and neonatal care in the Democratic Republic of the Congo: A study of the variability in user fees in Lubumbashi, 2014
Source: PLoS One. 2018 Oct 10;13(10):e0205082. doi: 10.1371/journal.pone.0205082 (PMC6179261; doi:10.1371/journal.pone.0205082)

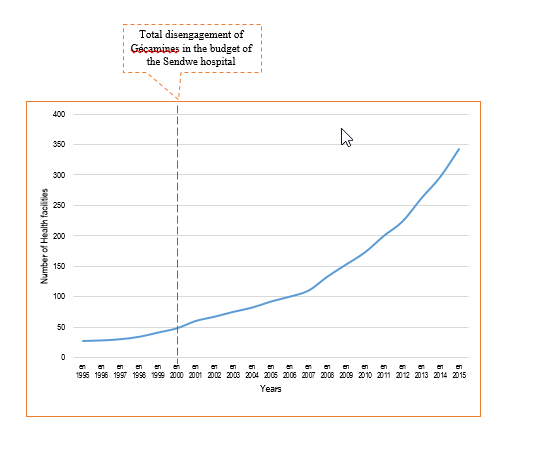

Supplement: S1 Fig — (TIF) [file pone.0205082.s002.tif]

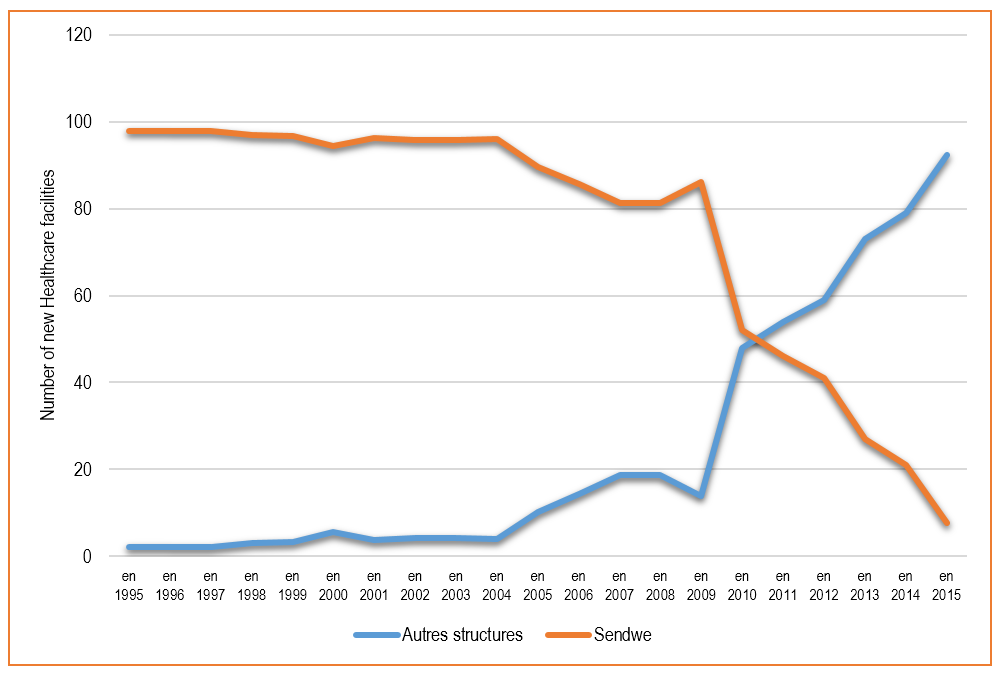

Supplement: S2 Fig — (TIF) [file pone.0205082.s003.tif]

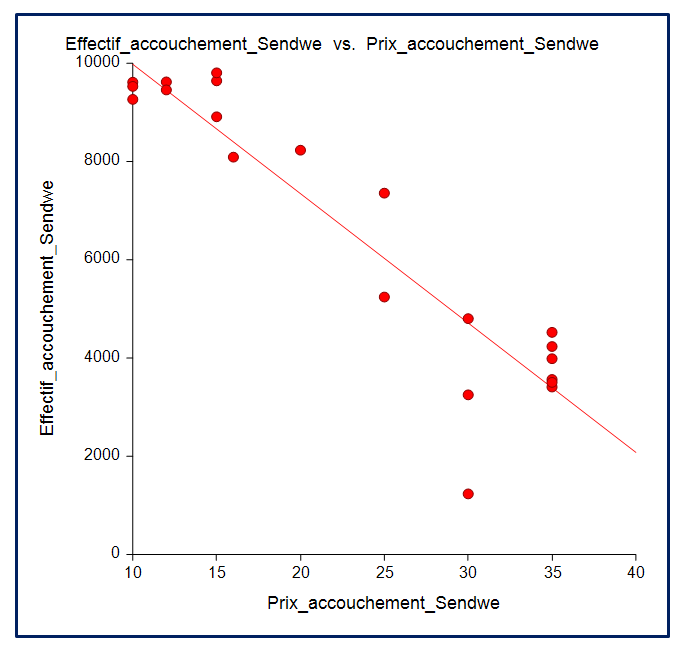

Supplement: S3 Fig — Coefficients of regression: -0.805; 95%IC: -0.521 to -1.00 P < 0,001. (TIF) [file pone.0205082.s004.tif]
