# Supplementary material for: Commercialization of obstetric and neonatal care in the Democratic Republic of the Congo: A study of the variability in user fees in Lubumbashi, 2014
Source: PLoS One. 2018 Oct 10;13(10):e0205082. doi: 10.1371/journal.pone.0205082 (PMC6179261; doi:10.1371/journal.pone.0205082)
Supplement: S1 Text — (DOC) [file pone.0205082.s005.doc]

**EXPENSES FOR OBSTETRIC AND NEONATAL CARE IN LUBUMBASHI: TOOL FOR DATA COLLECTION**

| **Characteristics of health facilities (HCF)** | **Answers** |
| --- | --- |
| **Health Zone** | **/................................/** |
| **Health center** | **/................................/** |
| **Type of health facility**  *Health Center = 1*  *General Hospital Reference = 2*  *General Provincial Hospital = 3*  *University Clinics = 4*  *Medical Center = 5*  *Clinic = 6* | **/................................/** |
| **Health Sector**  *STATE = 1*  *Company = 2*  *Private = 3*  *Denominational = 4* | **/................................/** |
| **Name of Health facility** | **/................................/** |
| **Code of parturient** | **/................................/** |
| **Date of admission of the parturient** | **/................................/** |

**I. General Information of women and their households**

| **[Q1]** | **[Q2]** | **[Q3]** | **[Q4]** | **[Q5]** | **[Q6]** | **[Q7]** | **[Q8]** | **[Q9]** |
| --- | --- | --- | --- | --- | --- | --- | --- | --- |
| What is your age (years)? | In which commune do you live??  *Katuba =1*  *Lubumbashi =2*  *Kenya =3*  *Kamalondo =4*  *Ruashi =5*  *Kampemba =6*  *Appendix =7* | What is your current marital status?  *Married = 1*  *Single = 2* | What is your education level?  *P1 = 1*  *P2 = 2*  *P3 = 3*  *P4 = 4*  *P5 = 5*  *P6 = 6*  *S1 = 7*  *S2 = 8*  *S3 = 9*  *S4 = 10*  *S5 = 11*  *S6 = 12*  *SU = 13* | What is your current occupation?  *Homemaking = 1*  *Vendor = 2*  *Agriculture = 3*  *State official = 4*  *Public company = 5*  *Private company = 6*  *Liberal occupation = 7*  *Pupil /Student = 8* | How old is your partner (husband)? | What is your husband's study level?  *P1 = 1*  *P2 = 2*  *P3 = 3*  *P4 = 4*  *P5 = 5*  *P6 = 6*  *S1 = 7*  *S2 = 8*  *S3 = 9*  *S4 = 10*  *S5 = 11*  *S6 = 12*  *SU = 13* | What is the current occupation of your husband?  *Homemaking = 1*  *Vendor = 2*  *Agriculture = 3*  *State official = 4*  *Public company = 5*  *Private company = 6*  *Liberal occupation = 7*  *Mining = 8*  *Pupil /Student = 9* | Who owns the house in which you are living?  *Landlord = 1*  *Owner = 2*  *Family home = 3*  *Do not know = 4* |
| /...../......./ | /........../ | /........../ | / ......... / | /.........../ | /...../......./ | /......./ | /.........../ | /.../.../..../ |

| **[Q10]** | **[Q11]** | **[Q12]** | **[Q13]** | **[Q14]** | **[Q15]** | **[Q16]** | **[Q17]** | **[Q18]** | | **[Q19]** |
| --- | --- | --- | --- | --- | --- | --- | --- | --- | --- | --- |
| What is your relationship with the head of household?  *Bride = 1*  *Sister = 2*  *Girl = 3*  *Little girl = 4*  *Responsible = 5* | How many people live in your household including you? | How many rooms has your household? | What types of materials are made the walls of your house?  *Cooked brick/Cement =1*  *Dobe brick =2*  *Clay = 3*  *Thatch = 4*  *Tarpaulins = 5* | What kind of material is made the roof of your house?  *Concrete = 1*  *Sheet /tile = 2*  *Thatch = 3*  *Sheeting /bags = 4* | What is the main source of water supply in your household?  *Tap = 1*  *Tank truck = 2*  *Wells dug = 3*  *Backwaters = 4*  *Kishimpo = 5*  *Wells drilled = 6* | What is the main source of lighting in your household?  *Public electricity = 1*  *Generator = 2*  *Rechargeable lamp = 3*  *Oil lamp = 4*  *Candles = 5*  *Lanterns = 6*  *Oil = 7*  *Other to specify = 8* | What is the main source of energy for food preparation in your household?  *Public Electricity / Gas =1*  *Charcoal =2*  *Buches =3*  *Thatch =4*  *Other to specify = 5* | Please, specify one of the following goods, those you have in your household?  *Present = 1*  *Absent =0* | | What type of toilet do you use in your household?  *Toilet flush =1*  *Turkish pit =2*  *With water jet = 3*  *Tank = 4*  *Moreover = 5*  *Other = 6* |
|  |  |  |  |  |  |  |  | Parcel |  |  |
|  |  |  |  |  |  |  |  | Freezer fridge |  |  |
|  |  |  |  |  |  |  |  | Color TV |  |  |
|  |  |  |  |  |  |  |  | DVD player |  |  |
|  |  |  |  |  |  |  |  | Phone |  |  |
|  |  |  |  |  |  |  |  | Car |  |  |
|  |  |  |  |  |  |  |  | Computer |  |  |
|  |  |  |  |  |  |  |  | Motorbike |  |  |
|  |  |  |  |  |  |  |  | Furniture |  |  |
|  |  |  |  |  |  |  |  | Farms |  |  |
|  |  |  |  |  |  |  |  | Cultivable land |  |  |
| / ...... / | / ...... / | /......./ | / ...... / | / ............ / | /............../ | / ............... / | /............./ | Bike |  | /........../ |

**2. Obstetrical History and Prenatal Consultations (PNC)**

| **[Q20]** | **[Q21]** | **[Q22]** | **[Q23]** | **[Q24]** | **[Q25]** | **[Q26]** | **[Q27]** | **[Q28]** |
| --- | --- | --- | --- | --- | --- | --- | --- | --- |
| How many times have you given birth in your life? | Did you attend the prenatal care (PNC) during your pregnancy?  *Yes = 1*  *No = 0* | If yes, give the name of the health facility in which you have followed the PNC. | What is your date of last menstrual period | If you had followed the PNC, at how many months of pregnancy did you start? | Can you give month of the start of your PNC? | How many times had you attended PNC? | Have you experienced a complication of your health condition during pregnancy?  *Yes = 1*  *No = 0* | If yes, which one?  *Antenatal hemorrhage = 1*  *Hypertension = 2*  *Diabetes = 3*  *Other = 4* |
| / ............ / | / ............ / | / ............................................... / | / .................. / | / ......... / | / ...................... / | / ............ / | / ............ / | / ............ / |
|  |  |  |  |  |  |  |  |  |
|  |  |  |  |  |  |  |  |  |
|  |  |  |  |  |  |  |  |  |

**3. Situation of the current delivery**

| **[Q29]** | **[Q30]** | **[Q31]** | **[Q32]** | **[Q33]** |  | **[Q34]** | **[Q35]** | **[Q36]** | **[Q37]** |
| --- | --- | --- | --- | --- | --- | --- | --- | --- | --- |
| Date of admission to this maternity | Reason for consultation  *Lombohypogastralgia (childbirth) = 1*  *Antenatal hemorrhage = 2*  *Rupture of the membranes = 3*  *No fetal movement =4*  *Convulsion = 5* | Delivery date | Complications at childbirth  *Eclampsia =1*  *Abruptio placentae = 2*  *Placenta previa =3*  *Obstructed labor=4*  *Hemorrhage = 5*  *Infection = 6*  *Uterine rupture =7*  *Other = 8* | Mode of delivery  *Vaginal delivery=0*  *Cesarean = 1* | Qualification of the Health care provider in case of vaginal delivery  *Nurse (e) = 1*  *Midwife = 2*  *General practionner = 3*  *Obstetrician = 4* | **If Caesarean**, Specify the date and time of the decision of the Caesarean. | **If Caesarean**, Indicate the date and time of surgery | **If Caesarean**, Staff qualifications who performed the intervention  *GP =1*  *Obstetrician =2* | **If Caesarean**  How did you get the surgical kit that was used for your surgery?  *Provided by maternity ward = 1*  *Purchased at the maternity ward = 2*  *Purchased at the market = 3* |
| /..................../ | / ............ / | /........................../ | / ......... / | /........../ | /........../ | /............................./ | /............................./ | /............./ | / ............ / |
|  |  |  |  |  |  |  |  |  |  |
|  |  |  |  |  |  |  |  |  |  |
|  |  |  |  |  |  |  |  |  |  |
|  |  |  |  |  |  |  |  |  |  |
|  |  |  |  |  |  |  |  |  |  |

**4. Information to look for in the registers of the maternity and fold of the parturient**

| **[Q38]** | | **[Q39]** | |
| --- | --- | --- | --- |
| Complications at childbirth  *Yes = 1*  *No = 0* | | Health interventions received by the woman and her newborn  *Yes = 1*  *No = 0* | |
| Preeclampsia/Eclampsia |  | Administration of parenteral antibiotics | /...................................../ |
| Abruptio placentae |  | Administration of uterotonic drugs | /...................................../ |
| Placenta previa |  | Controlled cord traction | /...................................../ |
| Obstructed labor |  | Uterine massage after removal of the placenta | /...................................../ |
| Hemorrhage |  | Administration of parenteral anticonvulsants for preeclampsia and eclampsia | /...................................../ |
| Infection |  | Manual removal of the placenta | /...................................../ |
| Uterine rupture |  | Instrumental evacuation of the placenta (remove retained products) | /...................................../ |
| Other |  | Assisted vaginal delivery by ventouse | /...................................../ |
|  |  | Resuscitation of the newborn by mask | /...................................../ |
|  |  | Cesarean section | /...................................../ |
|  |  | Oxytocin infusion | /...................................../ |
|  |  | Blood transfusion (mother) | /...................................../ |
|  |  | Management of other obstetric emergencies | /...................................../ |
|  |  | Thermal care of the newborn in the incubator | /...................................../ |
|  |  | Kangaroo Thermal Care | /...................................../ |
|  |  | Blood transfusion (newborn) | /...................................../ |
|  |  | Phototherapy | /...................................../ |
|  |  | Care of the sick newborn | /...................................../ |
|  |  | Oxygen therapy of the newborn | /...................................../ |
|  |  | Feeding the sick newborn by nasogastric tube | /...................................../ |
|  |  | Antibiotherapy of the newborn | /...................................../ |

5. MONITORING OF EXPENSES RELATED TO DELIVERY

**Check, in the woman's file, the health interventions offered to women and newborns during their stay at the maternity ward**

**Also ask the head of the maternity or midwife to confirm the following expenses incurred by the woman**

**[Q40] How much did you pay for each of these aspects of care (FC)?**

|  | **Day_1** | **Day_2** | **Day_3** | **Day_4** | **Day_5** | **Day_6** | **Day_7** | **Day_8** | **Day_9** |
| --- | --- | --- | --- | --- | --- | --- | --- | --- | --- |
| Maternity Sheet |  |  |  |  |  |  |  |  |  |
| Equipment (gloves…) |  |  |  |  |  |  |  |  |  |
| Normal delivery (act) |  |  |  |  |  |  |  |  |  |
| Childbirth obstructed (act) |  |  |  |  |  |  |  |  |  |
|  |  |  |  |  |  |  |  |  |  |
| **MOTHER** |  |  |  |  |  |  |  |  |  |
| Scheduled caesarean section |  |  |  |  |  |  |  |  |  |
| Emergency Caesarean |  |  |  |  |  |  |  |  |  |
| Other surgeries |  |  |  |  |  |  |  |  |  |
| Surgery kit |  |  |  |  |  |  |  |  |  |
| **Care after childbirth** |  |  |  |  |  |  |  |  |  |
| Stay |  |  |  |  |  |  |  |  |  |
| Meal |  |  |  |  |  |  |  |  |  |
| Wound dressing |  |  |  |  |  |  |  |  |  |
| Episiotomy |  |  |  |  |  |  |  |  |  |
| Blood transfusion |  |  |  |  |  |  |  |  |  |
| curettage |  |  |  |  |  |  |  |  |  |
| Manual uterine revision |  |  |  |  |  |  |  |  |  |
| Oxygen therapy |  |  |  |  |  |  |  |  |  |
| Oxytocin infusion |  |  |  |  |  |  |  |  |  |
| Magnesium Sulfate Injection |  |  |  |  |  |  |  |  |  |
|  |  |  |  |  |  |  |  |  |  |
| **NEWBORN** |  |  |  |  |  |  |  |  |  |
| Oxygen therapy |  |  |  |  |  |  |  |  |  |
| Phototherapy |  |  |  |  |  |  |  |  |  |
| General care of the newborn |  |  |  |  |  |  |  |  |  |
| Blood transfusion |  |  |  |  |  |  |  |  |  |
| Thermal care in the incubator |  |  |  |  |  |  |  |  |  |
| Mask ventilation |  |  |  |  |  |  |  |  |  |
| Perfusion |  |  |  |  |  |  |  |  |  |
| Stay in neonatology |  |  |  |  |  |  |  |  |  |
|  |  |  |  |  |  |  |  |  |  |
| **AGGREGATE AMOUNT** |  |  |  |  |  |  |  |  |  |
| Vaginal delivery |  |  |  |  |  |  |  |  |  |
| Cesarean Section |  |  |  |  |  |  |  |  |  |

**[Q40] How much did you pay for each aspect of care (FC)?**

|  | **Day_10** | **Day_11** | **Day_12** | **Day_13** | **Day_14** | **Day_15** | **Day_16** | **Day_17** | **Day_18** |
| --- | --- | --- | --- | --- | --- | --- | --- | --- | --- |
| Maternity Sheet |  |  |  |  |  |  |  |  |  |
| Equipment (gloves…) |  |  |  |  |  |  |  |  |  |
| Normal delivery (act) |  |  |  |  |  |  |  |  |  |
| Childbirth obstructed (act) |  |  |  |  |  |  |  |  |  |
|  |  |  |  |  |  |  |  |  |  |
| **MOTHER** |  |  |  |  |  |  |  |  |  |
| Scheduled caesarean section |  |  |  |  |  |  |  |  |  |
| Emergency Caesarean |  |  |  |  |  |  |  |  |  |
| Other surgeries |  |  |  |  |  |  |  |  |  |
| Surgery kit |  |  |  |  |  |  |  |  |  |
| **Care after childbirth** |  |  |  |  |  |  |  |  |  |
| Stay |  |  |  |  |  |  |  |  |  |
| Meal |  |  |  |  |  |  |  |  |  |
| Wound dressing |  |  |  |  |  |  |  |  |  |
| Episiotomy |  |  |  |  |  |  |  |  |  |
| Blood transfusion |  |  |  |  |  |  |  |  |  |
| curettage |  |  |  |  |  |  |  |  |  |
| Manual uterine revision |  |  |  |  |  |  |  |  |  |
| Oxygen therapy |  |  |  |  |  |  |  |  |  |
| Oxytocin infusion |  |  |  |  |  |  |  |  |  |
| Magnesium Sulfate Injection |  |  |  |  |  |  |  |  |  |
|  |  |  |  |  |  |  |  |  |  |
| **NEWBORN** |  |  |  |  |  |  |  |  |  |
| Oxygen therapy |  |  |  |  |  |  |  |  |  |
| Phototherapy |  |  |  |  |  |  |  |  |  |
| General care of the newborn |  |  |  |  |  |  |  |  |  |
| Blood transfusion |  |  |  |  |  |  |  |  |  |
| Thermal care in the incubator |  |  |  |  |  |  |  |  |  |
| Mask ventilation |  |  |  |  |  |  |  |  |  |
| Perfusion |  |  |  |  |  |  |  |  |  |
| Stay in neonatology |  |  |  |  |  |  |  |  |  |
|  |  |  |  |  |  |  |  |  |  |
| **AGGREGATE AMOUNT** |  |  |  |  |  |  |  |  |  |
| Vaginal delivery |  |  |  |  |  |  |  |  |  |
| Cesarean Section |  |  |  |  |  |  |  |  |  |

**[Q40] How much did you pay for each aspect of care (FC)**?

|  | **Day_19** | **Day_20** | **Day_21** | **Day_22** | **Day_23** | **Day_24** | **Day_25** | **Day_26** | **Day_27** |
| --- | --- | --- | --- | --- | --- | --- | --- | --- | --- |
| Maternity Sheet |  |  |  |  |  |  |  |  |  |
| Equipment (gloves…) |  |  |  |  |  |  |  |  |  |
| Normal delivery (act) |  |  |  |  |  |  |  |  |  |
| Childbirth obstructed (act) |  |  |  |  |  |  |  |  |  |
|  |  |  |  |  |  |  |  |  |  |
| **MOTHER** |  |  |  |  |  |  |  |  |  |
| Scheduled caesarean section |  |  |  |  |  |  |  |  |  |
| Emergency Caesarean |  |  |  |  |  |  |  |  |  |
| Other surgeries |  |  |  |  |  |  |  |  |  |
| Surgery kit |  |  |  |  |  |  |  |  |  |
| **Care after childbirth** |  |  |  |  |  |  |  |  |  |
| Stay |  |  |  |  |  |  |  |  |  |
| Meal |  |  |  |  |  |  |  |  |  |
| Wound dressing |  |  |  |  |  |  |  |  |  |
| Episiotomy |  |  |  |  |  |  |  |  |  |
| Blood transfusion |  |  |  |  |  |  |  |  |  |
| curettage |  |  |  |  |  |  |  |  |  |
| Manual uterine revision |  |  |  |  |  |  |  |  |  |
| Oxygen therapy |  |  |  |  |  |  |  |  |  |
| Oxytocin infusion |  |  |  |  |  |  |  |  |  |
| Magnesium Sulfate Injection |  |  |  |  |  |  |  |  |  |
|  |  |  |  |  |  |  |  |  |  |
| **NEWBORN** |  |  |  |  |  |  |  |  |  |
| Oxygen therapy |  |  |  |  |  |  |  |  |  |
| Phototherapy |  |  |  |  |  |  |  |  |  |
| General care of the newborn |  |  |  |  |  |  |  |  |  |
| Blood transfusion |  |  |  |  |  |  |  |  |  |
| Thermal care in the incubator |  |  |  |  |  |  |  |  |  |
| Mask ventilation |  |  |  |  |  |  |  |  |  |
| Perfusion |  |  |  |  |  |  |  |  |  |
| Stay in neonatology |  |  |  |  |  |  |  |  |  |
|  |  |  |  |  |  |  |  |  |  |
| **AGGREGATE AMOUNT** |  |  |  |  |  |  |  |  |  |
| Vaginal delivery |  |  |  |  |  |  |  |  |  |
| Cesarean Section |  |  |  |  |  |  |  |  |  |

**[Q40] How much did you pay for each aspect of care (FC)**?

|  | **Day_28** | **Day_29** | **Day_30** | **Day_31** | **Day_32** | **Day_33** | **Day_34** | **Total** |
| --- | --- | --- | --- | --- | --- | --- | --- | --- |
| Maternity Sheet |  |  |  |  |  |  |  |  |
| Equipment (gloves…) |  |  |  |  |  |  |  |  |
| Normal delivery (act) |  |  |  |  |  |  |  |  |
| Childbirth obstructed (act) |  |  |  |  |  |  |  |  |
|  |  |  |  |  |  |  |  |  |
| **MOTHER** |  |  |  |  |  |  |  |  |
| Scheduled caesarean section |  |  |  |  |  |  |  |  |
| Emergency Caesarean |  |  |  |  |  |  |  |  |
| Other surgeries |  |  |  |  |  |  |  |  |
| Surgery kit |  |  |  |  |  |  |  |  |
| **Care after childbirth** |  |  |  |  |  |  |  |  |
| Stay |  |  |  |  |  |  |  |  |
| Meal |  |  |  |  |  |  |  |  |
| Wound dressing |  |  |  |  |  |  |  |  |
| Episiotomy |  |  |  |  |  |  |  |  |
| Blood transfusion |  |  |  |  |  |  |  |  |
| curettage |  |  |  |  |  |  |  |  |
| Manual uterine revision |  |  |  |  |  |  |  |  |
| Oxygen therapy |  |  |  |  |  |  |  |  |
| Oxytocin infusion |  |  |  |  |  |  |  |  |
| Magnesium Sulfate Injection |  |  |  |  |  |  |  |  |
|  |  |  |  |  |  |  |  |  |
| **NEWBORN** |  |  |  |  |  |  |  |  |
| Oxygen therapy |  |  |  |  |  |  |  |  |
| Phototherapy |  |  |  |  |  |  |  |  |
| General care of the newborn |  |  |  |  |  |  |  |  |
| Blood transfusion |  |  |  |  |  |  |  |  |
| Thermal care in the incubator |  |  |  |  |  |  |  |  |
| Mask ventilation |  |  |  |  |  |  |  |  |
| Perfusion |  |  |  |  |  |  |  |  |
| Stay in neonatology |  |  |  |  |  |  |  |  |
|  |  |  |  |  |  |  |  |  |
| **AGGREGATE AMOUNT** |  |  |  |  |  |  |  |  |
| Vaginal delivery |  |  |  |  |  |  |  |  |
| Cesarean Section |  |  |  |  |  |  |  |  |

**6. Childbirth planning, care financing**

| **[Q41]** | **[Q42]** | **[Q43]** | **[Q44]** | **[Q45]** | **[Q46]** | **[Q47]** | **[Q48]** | **[Q49]** | **[Q50]** |
| --- | --- | --- | --- | --- | --- | --- | --- | --- | --- |
| Had you planned to give birth in the maternity ward?  *Yes = 1*  *No = 0* | If not, in what maternity ward did you plan to give birth? | Why did you decide to give birth in the current maternity ward?  *Cheaper = 1*  *Own = 2*  *friendly staff = 3*  *Near = 4*  *No choice = 5*  *Subscriber = 6*  *Referred = 7* | Did you know in advance how much money you should pay for your maternity care?  *Yes = 1*  *No = 0* | If so, how did you know?  *Information to CPN = 1*  *Information from neighboring = 2* | How did you appreciate the price of care in this maternity ward?  *Dear = 1*  *Cheapest = 2* | Who paid for your care here at the maternity ward?  *Spouse = 1*  *Parents = 2*  *Myself = 3*  *NGOs = 4*  *Church = 5*  *Sibling = 6*  *Subscriber = 7* | **If your spouse or yourself,** how did you raise all the money required for your care?  *Salary = 1*  *Support by the husband's company = 2*  *Sale of household goods = 3*  *My savings = 4*  *Discount = 5* | By what means of transport did you arrive here at the maternity ward?  *Ambulance = 1*  *Bus = 2*  *Taxi = 3*  *Personal car = 4*  *Bike = 5*  *Motorcycle = 6*  *Feet = 7* | If other means than the feet and the personal vehicle,  How much money have you paid for your transport so far to the maternity ward? |
| / ...... / | / ...................... / | / ...... / | / ......... / | / ...... / | / ......... / | / ...... / | / ...... / | /........../ | / ...... ………/ |

|  |  |  |  |  | **TRANSFER** | | | |
| --- | --- | --- | --- | --- | --- | --- | --- | --- |
| **[Q51]** | **[Q52]** | **[Q53]** | **[Q54]** | **[Q55]** | **[Q56]** | **[Q57]** | **[Q58]** | **[Q59]** |
| If by vehicle, was it individual or in common?  *Individual = 1*  *Common = 2* | How much (FC) will you pay to go home to home? | Have you been accompanied and kept at the maternity ward by a relative?  *Yes = 1*  *No = 0* | If so, what relationship there is between you and that person?  *Mother = 1*  *Sister-in law = 2*  *Friend = 3*  *Mother-in law = 4*  *Sister = 5*  *Spouse = 6* | What is his usual main occupation?  *Homemaking = 1*  *Vendor = 2*  *Other: ...* | Have you been referred by another health facility or you have consulted directly in it?  *Direct consultation = 1*  *Reference = 2* | If reference, what is the reason for this reference?  *Hypertension = 1*  *Convulsion = 2*  *Fever = 3*  *Hemorrhage = 4*  *Obstructed Labor = 5*  *Absence of fetal movements = 6* | How much money did you pay for care received in the first structure (CF)? | By what means of transport have you left this health facility so far?  *Ambulance = 1*  *Bus = 2*  *Taxi = 3*  *Personal car = 4*  *Bike = 5*  *Motorcycle = 6*  *Feet = 7* |
| / ............ / | / ................. / | / ......... / | / ............ / | / ............... / | / ............... / | / ............... / | / ............... / | / ............... / |

| **TRANSFER** | | | | |  |
| --- | --- | --- | --- | --- | --- |
| **[Q60]** | **[Q61]** | **[Q62]** | **[Q63]** | **[Q64]** | **[Q65]** |
| If by bicycle, motorcycle, transportation or ambulance, how much did you pay for this transport? | Have you been accompanied by a health worker during this reference?  *Yes = 1*  *No = 0* | Did you arrive here on the same reference day?  *Yes = 1*  *No = 0* | If not, where were you before coming here?  *In the same health facility =1*  *At home = 2* | Why did not you arrive here the same day?  *To pay the expenses of the first structure = 1*  *Lack of transport = 2* | If you happen to have another pregnancy, would you agree to return to give birth in this Health facility?  *Yes = 1*  *No = 0* |
| / ............... / | / ............ / | / ......... / | / ............ / | / ............... / | / ............ / |

*Lubumbashi ...... ../ ...... ../ 2014*
